# A new specimen of *Manchurochelys manchoukuoensis* from the Early Cretaceous Jehol Biota of Chifeng, Inner Mongolia, China and the phylogeny of Cretaceous basal eucryptodiran turtles

**DOI:** 10.1186/1471-2148-14-77

**Published:** 2014-04-05

**Authors:** Chang-Fu Zhou, Márton Rabi, Walter G Joyce

**Affiliations:** 1 Paleontological Institute, Shenyang Normal University, 253 North Huanghe Street, Shenyang, Liaoning 110034, People’s Republic of China; 2 Institut für Geowissenschaften, University of Tübingen, Hölderlinstraße 12, Tübingen 72074, Germany; 3 Department of Paleontology & MTA – ELTE Lendület Dinosaur Research Group, Eötvös Loránd University, Budapest, Hungary; 4 Department of Geosciences, University of Fribourg, Fribourg 1700, Switzerland

## Abstract

**Background:**

*Manchurochelys manchoukuoensis* is an emblematic turtle from the Cretaceous Yixian Formation of Liaoning, China, a geological rock unit that is famous for yielding perfectly preserved skeletons of fossil vertebrates, including that of feathered dinosaurs. *Manchurochelys manchoukuoensis* was one of the first vertebrates described from this fauna, also known as the Jehol Biota. The holotype was lost during World War II and only one additional specimen has been described since. *Manchurochelys manchoukuoensis* is a critical taxon for unraveling the phylogenetic relationships of Cretaceous pancryptodires from Asia, a group that is considered to be of key importance for the origin of crown-group hidden-neck turtles (Cryptodira).

**Results:**

A new specimen of *Manchurochelys manchoukuoensis* is described here from the Jiufotang Formation of Qilinshan, Chifeng, Inner Mongolia, China. This is the third specimen described and expands the range of this taxon from the Yixian Formation of the Fuxin-Yixian Basin in Liaoning to the Jiufotang Formation of the Chifeng-Yuanbaoshan Basin. A possible temporal extension of the range is less certain. The new finding adds to our understanding of the morphology of this taxon and invites a thorough revision of the phylogeny of Macrobaenidae, Sinemydidae, and closely allied forms.

**Conclusions:**

Our comprehensive phylogenetic analyses of Cretaceous Asian pancryptodires yielded two main competing hypotheses: in the first these taxa form a paraphyletic grade, whereas in the second they form a monophyletic clade. The inclusion of problematic tree changing taxa, such as Panpleurodires (stem + crown side-neck turtles) has a major influence on the phylogenetic relationships of Sinemydidae and closely allied forms. *Manchurochelys manchoukuoensis* nests within Sinemydidae together with *Sinemys* spp. and *Dracochelys bicuspis* in the majority of our analyses.

## Background

To date, three turtle taxa have been recognized in the Early Cretaceous Jehol Biota of western Liaoning and adjacent areas: *Manchurochelys manchoukuoensis* Endo and Shikama 1942 [1]; *Ordosemys liaoxiensis* (Ji 1995) [2,3]; and *Liaochelys jianchangensis* Zhou 2010 [4]. Of these, *M. manchoukuoensis* is notable because it was one of the first tetrapod fossils to be described from the Jehol Biota, together with the choristodere *Manchurosuchus splendens* and the lizard *Yabeinosaurus tenuis*. Unfortunately, the holotype, a partial shell, appears to have been lost during World War II [5]. Our knowledge regarding the anatomy of this species was nevertheless recently expanded by the referral of a second specimen, which consists of a nearly complete skeleton [5], but much remains to be learned about this taxon, in particular in regards to its skeletal anatomy, phylogenetic relationships, and its geographic and temporal distribution.

**Figure 1:**
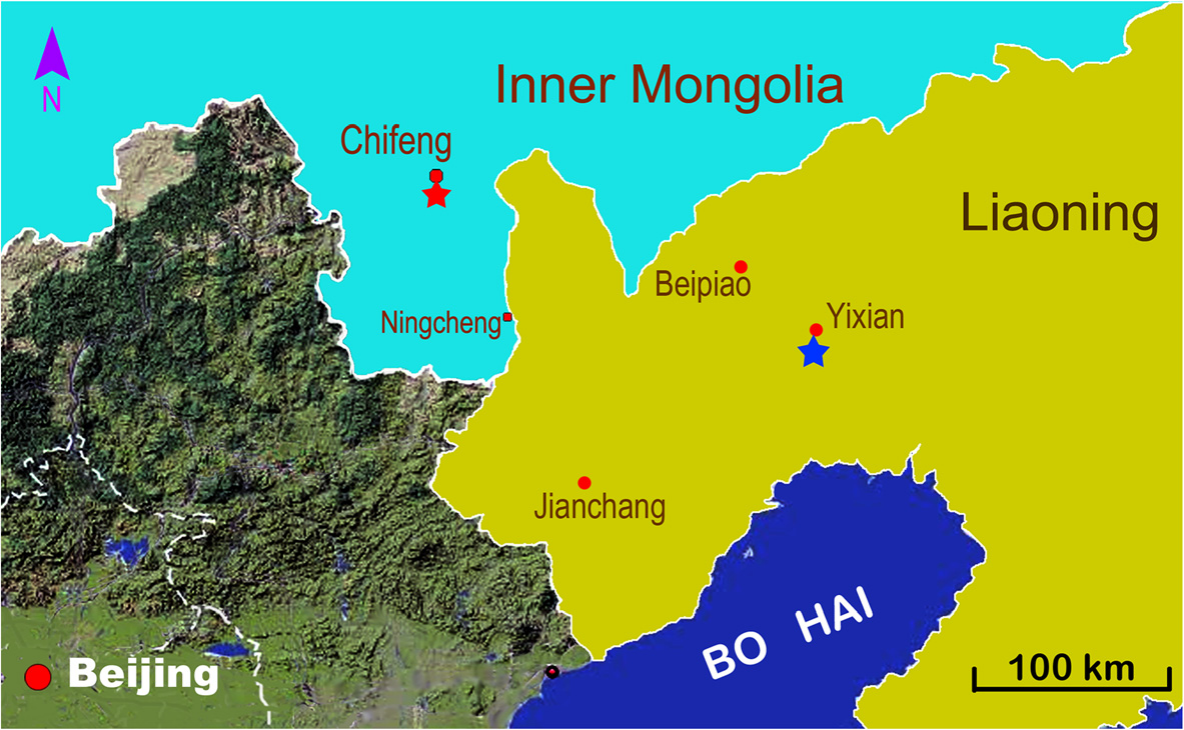
**Map showing the known localities of *Manchurochelys manchoukuoensis*, in Qilinshan (=Heishangou; marked by a red asterisk; E118°50′46.4″, N42°08′33.3″), Chifeng City, Inner Mongolia; and in Yixian (marked by a blue asterisk), Jinzhou City, Liaoning Province.**

In the present paper, a new partial skeleton of *M. manchoukuoensis* is described from a new site in the Jiufotang Formation of Qilinshan, Chifeng, Inner Mongolia (Figure 1). In addition to expanding the geographical distribution of *M. manchoukuoensis* to Inner Mongolia, this specimen is interesting because it allows a reassessment of the morphology and phylogenetic relationships of this enigmatic species.

Asian Cretaceous basal eucryptodires, such as *M. manchoukuoensis*, are widely recognized as a critical group for resolving the early evolution of pancryptodires [6-20], a clade that represents about 75% of extant turtle diversity. There currently is no consensus on the phylogenetic arrangement of Cretaceous pancryptodires, but most workers historically distinguished two primary groups with doubtful monophyly: Sinemydidae and Macrobaenidae [6,8-10,21-26]. There historically also was little agreement on the content of these taxa, but recent phylogenetic definitions provided some level of nomenclatural stability [27]. In particular, Sinemydidae is now defined as referring to the most inclusive clade containing *Sinemys lens* but not *Xinjiangchelys junggarensis, Macrobaena mongolica*, or any species of recent turtle, whereas Macrobaenidae is defined as referring to the most inclusive clade containing *Macrobaena mongolica* but not *Xinjiangchelys junggarensis*, *Sinemys lens*, or any species of Recent turtle. A summary of the taxonomic history of these groups is provided in Rabi et al. [19,27].

There have been numerous attempts to resolve the phylogeny and position of Macrobaenidae and Sinemydidae, but these either suffered from low taxon sampling and/or lack of specific characters and/or use of literature based data rather than actual observations on fossil specimens [4,5,11,12,14,17,18,20],[28-36]. Here, we considerably improve upon previous analyses by rescoring taxa based on our own observations of specimens, by adding six new taxa and new characters, and by testing for tree changing and wildcard taxa.

## Methods

The fossil described herein is housed in the Paleontological Museum of Liaoning (= Liaoning Paleontological Museum, PMOL), Shenyang Normal University, with the number PMOL-AR00180. The specimen was obtained in two blocks that were subsequently glued together during the preparation process at PMOL. The surrounding sediment was then removed to expose the skeleton in dorsal and ventral views.

The following fossil taxa were studied first hand for comparative purposes and for the phylogenetic analysis: *Dracochelys bicuspis* Gaffney and Ye, 1992 [8] (IVPP V4075 holotype); *Kirgizemys* (= *Hangaiemys*) *hoburensis* (Sukhanov and Narmandakh, 1974) [6,15] (PIN 3334-4, PIN 3334-1, PIN 3334-5, PIN 3334-16, PIN 3334-34, PIN 3334-35, PIN 3334-36, PIN 3334-37); *Judithemys sukhanovi* Parham and Hutchison, 2003 [14] (TMP 87.2.1 holotype); *Liaochelys jianchangensis* Zhou, 2010 [4] (PMOL-AR00140 holotype, PMOL-AR00160); *Manchurochelys manchoukuoensis* Endo and Shikama, 1942 [1] (PMOL AR00008); *Ordosemys leios*[10] (IVPP V9534-1 holotype, and material listed in Brinkman and Peng 1993 [10]); *Sinemys gamera* Brinkman and Peng 1993 [9] (IVPP V9532-1 holotype, IVPP V9532-11 and the material listed in Brinkman and Peng 1993 [9]); *Sinemys brevispinus* Tong and Brinkman, 2013 [37] (IVPP V9538-1 holotype); Wiman, 1930 [38] (IVPP V8755, IVPP V9533-1).

The cranial carotid circulation nomenclature follows Rabi et al. [36] and taxonomic nomenclature follows the phylogenetic definitions of Rabi et al. [27].

### Phylogenetic analysis

Four separate phylogenetic analyses were run in order to test the relationships of Cretaceous basal eucryptodires from Asia and North America. All analyses used a modified version of the latest global turtle character-taxon matrix by Rabi et al. [36], which in turn is based on Rabi et al. [27], Sterli and de la Fuente [20,23], Sterli [30], and Joyce [18]. In addition to the taxa sampled in Rabi et al. [36], the matrix includes *Liaochelys jianchangensis*, *Changmachelys bohlini* Brinkman et al., 2013 [35], *Sinemys gamera*, *Sinemys lens*, *Sinemys brevispinus*, and the skull of *Ordosemys* sp. [12]. The taxon *Ordosemys leios* is only considered to consist of material described in Brinkman and Peng [10]. *Manchurochelys manchoukuoensis* was scored on the basis of three specimens: the specimen described herein (PMOL-AR00180), the one described by Zhou [5]; PMOL-AR00008), and the lost holotype [1]. Several scorings were changed for *Kirgizemys hoburensis*, *Sinemys lens*, *Dracochelys bicuspis*, and *Ordosemys leios*, among others, based on personal observations of the relevant material (see Appendix 1 for list of changes).

The following characters were treated as ordered: 7 (Nasal A), 19 (Parietal H), 27 (Squamosal C), 40 (Maxilla D), 42 (Vomer A), 50 (Quadrate B + C), 52 (Antrum Postoticum A), 59 (Pterygoid B), 81 (Opisthotic C), 82 (Opisthotic D), 89 (Stapedial Artery B), 98 (Canalis Caroticum F), 120 (Carapace A), 121 (Carapace B), 130 (Peripheral A), 133 (Costal B), 138 (Supramarginal A), 158 (Hyoplastron B), 159 (Mesoplastron A), 161 (Hyoplastron B), 176 (Abdominal A), 213 (Cleithrum A), 214 (Scapula A), 232 (Manus B), 233 (Manus C). *Sphenodon punctatus*, *Owenetta kitchingorum*, *Simosaurus gaillardoti*, and *Anthodon serrarius* were designated as outgroups.

In each analysis we omitted the following characters: Maxilla B, Basioccipital B, Pterygoid M, and Cervical Vertebra D and K. Maxilla B was omitted because we cannot reproduce the meaning or scoring of this character as provided by Sterli and de la Fuente [20]. As scored, this character does not show any variation within Cretaceous basal eucryptodires and we therefore do not expect any impact from its omission.

Basioccipital B is omitted for similar reasons: the definition of a deep, C-shaped concavity on the basioccipital is quite vague since almost all turtles with basioccipital tubera have some sort of C-shaped concavity, but were scored as absent by Sterli and de la Fuente.

Pterygoid M is omitted because, unlike as stated [20], the derived state of this character (basisphenoid and pterygoid in different levels) is present in many basal taxa (actually being the ancestral state for turtles, e.g. *Proganochelys quenstedti*) and therefore the character should be rescored in the future.

Cervical D is omitted once again because we cannot reproduce the meaning of ‘triangular diapophysis’ and because the current distribution of this character does not help us either (scored as present for panpleurodires, *Chubutemys copelloi*, *Glyptops plicatulus* and baenids).

Finally, Cervical vertebra K is omitted because we find it redundant with Cervical Vertebra B (both characters pertain to the depth of the ventral keel on posterior cervicals).

The taxon-character matrix, the TNT file and strict consensus trees are deposited on the website of the journal as Additional files 1, 2 and 3 and in TreeBase (Study Accession URL: http://purl.org/phylo/treebase/phylows/study/TB2:S15457).

#### Analysis A

For this analysis, a simple heuristic search was performed in TNT [39,40] using the tree-bisection-reconnection swapping algorithm with thousands of random addition sequence replicates and 10 trees saved per replicate. Wildcard taxa were removed following the search to improve resolution within the strict consensus tree.

#### Analysis B

The protocol from ‘Analysis A’ was repeated, but this time the relationship of the major crown-cryptodire clades (not only Durocryptodira as in Rabi et al. [36]) were constrained following the current molecular consensus [41]: (Trionychia (Emydidae (Geoemydidae + Testudinidae)) + (Chelonioidea (Chelydridae + Kinosternoidea)))). The internal relationships of these clades were left unconstrained and *Platysternon megacephalum* was considered a stem-emydid. Heuristic searches were repeated until the most parsimonious trees (MPT) were found 30 times during each replicate (using the command “xmult = hits 30”).

#### Analysis C

The protocol from ‘Analysis B’ was repeated, but nine new characters that are thought to be relevant for the interrelationships of Cretaceous basal eucryptodires were added (see Appendix 2 for character definitions). Heuristic searches were repeated until the most parsimonious trees (MPT) were found 30 times during each replicate.

#### Analysis D

This analysis differs from ‘C’ in that *Basilochelys macrobios* and most pan-pleurodires except for *Podocnemis expansa* and *Pelomedusa subrufa* were excluded a priori before running the heuristic search. This experimental approach is justified by the work of Rabi et al. [27,36] in which the position of pan-pleurodires proved to be problematic in that xinjiangchelyids, sinemydids, and other, widely recognized Mesozoic stem-cryptodires were unorthodoxly placed outside of Testudines and in that Cryptodira was not found to be monophyletic relative to Pleurodira. As such, we were interested in testing how the removal of most pan-pleurodires affects tree topology, especially in the case of Mesozoic basal eucryptodires. The search was again repeated until the most parsimonious trees (MPT) were found 30 times during each replicate.

### Systematic Paleontology

TESTUDINATA Klein [42]

TESTUDINES Batsch [43]

PANCRYPTODIRA Joyce, Parham, and Gauthier [44]

**Figure 2:**
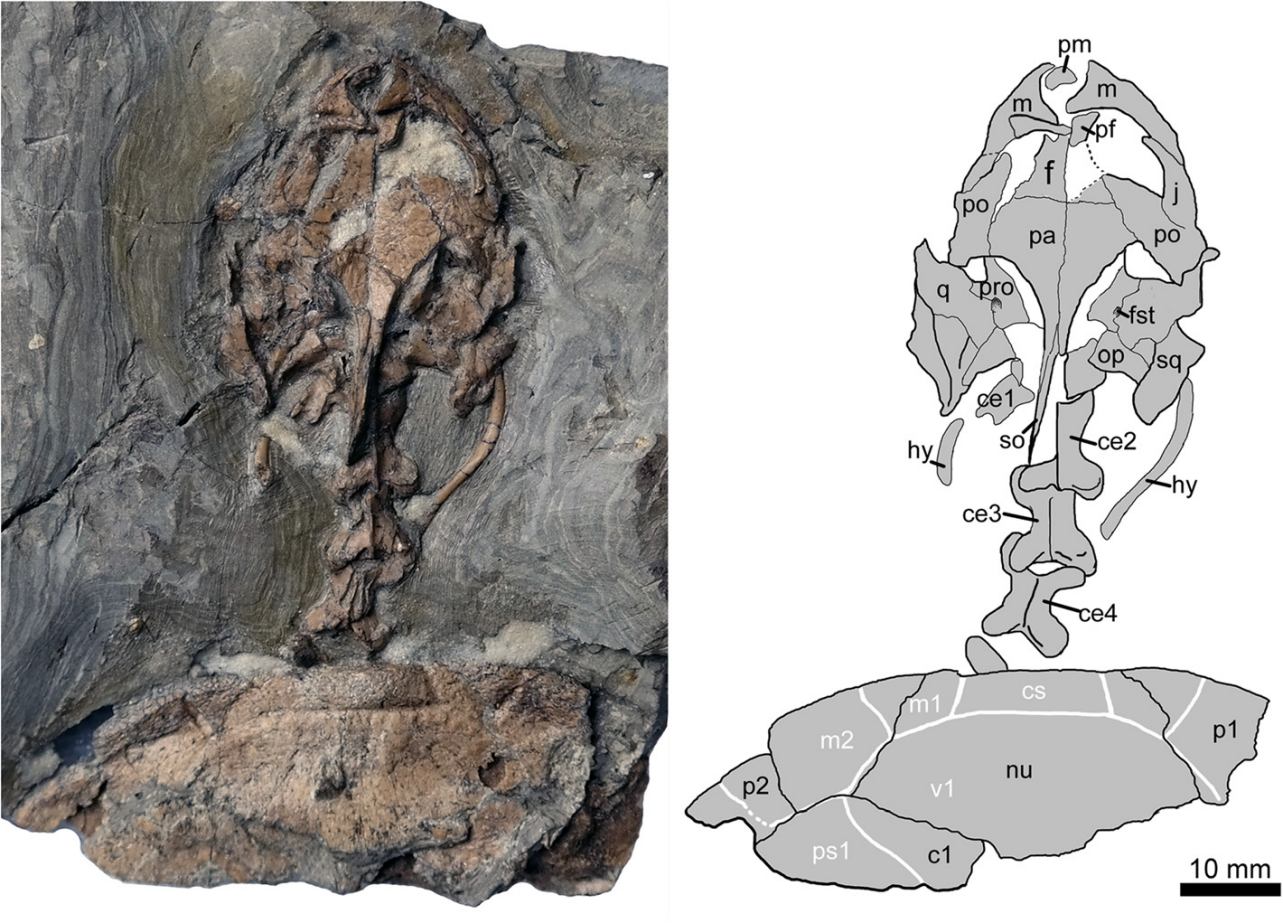
**New material of *Manchurochelys manchoukuoensis *(PMOL-AR00180) in dorsal view, from the Early Cretaceous Jiufotang Formation of Qilinshan, Chifeng, Inner Mongolia, China. Abbreviations: c1,** costal Plate 1; **cs,** cervical scale; **ce1-4**, cervical vertebrae 1-4; **f,** frontal; **fst,** foramen stapedio-temporale; **hy,** hyoid; **m,** maxilla; **m1-2,** marginal scales 1–2; **nu,** nuchal; **op,** opisthotic; **p1-2,** peripheral Plates 1–2; **pa,** parietal; **pf**, prefrontal; **pm,** premaxilla; **po,** postorbital; **pro,** prootic; **ps1,** pleural scale 1; **q,** quadrate; **so,** supraoccipital; **sq,** squamosal; **v1,** vertebral scale 1.

**Figure 3:**
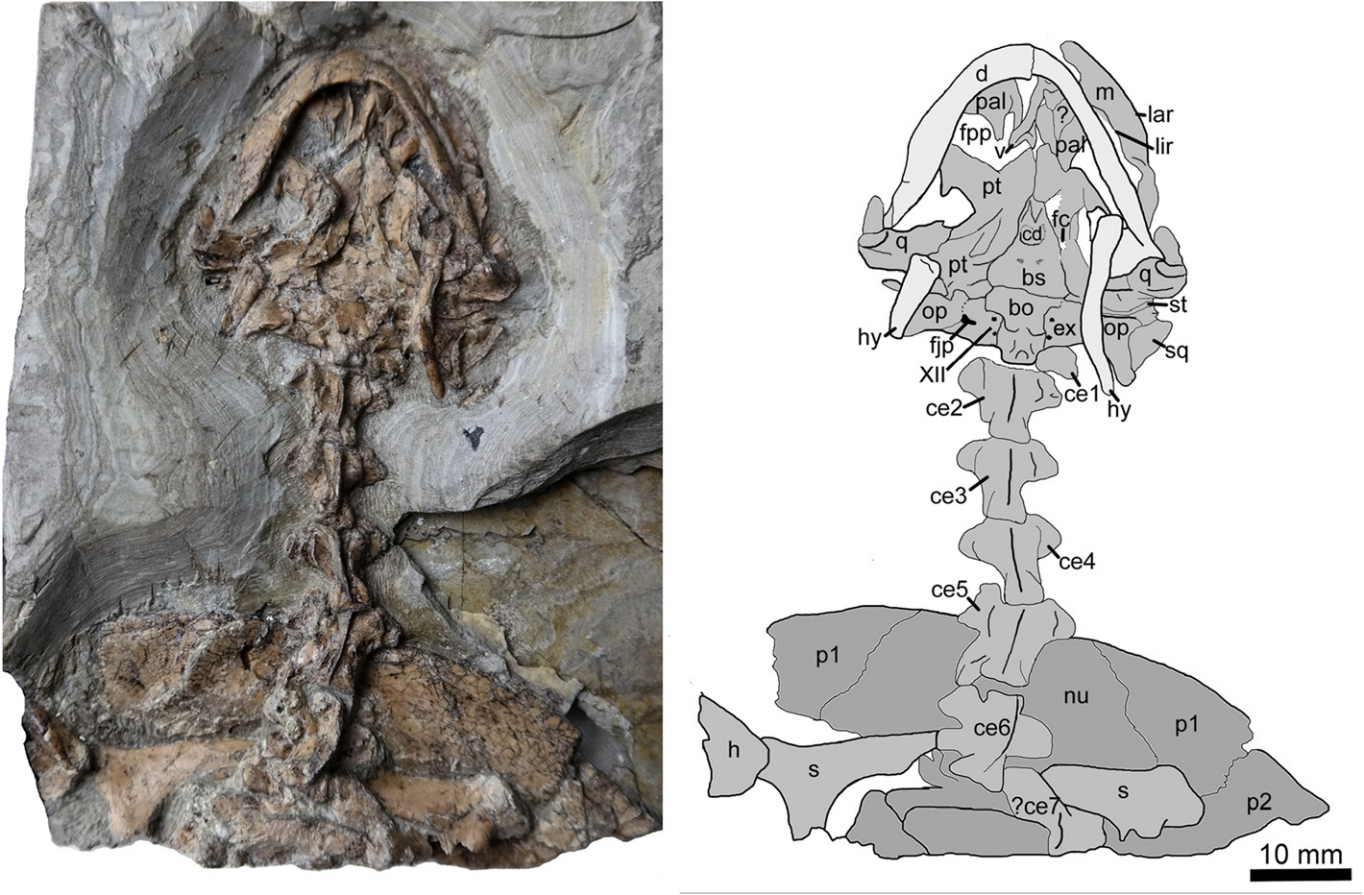
**New material of *Manchurochelys manchoukuoensis *(PMOL-AR00180) in ventral view, from the Early Cretaceous Jiufotang Formation of Qilinshan, Chifeng, Inner Mongolia, China. Abbreviations: bo,** basioccipital; **bs,** basisphenoid; **cd,** central depression of basisphenoid (maybe taphonomic); **ce1-7,** cervical vertebrae 1-7; **d,** dentary; **ex,** exoccipital; **fc**, fenestra caroticus; **fjp,** foramen jugulare posterius; **h,** humerus; **hy,** hyoid; **lar,** labial ridge of the triturating surface in maxilla; **lir,** lingual ridge of the triturating surface in maxilla; **m,** maxilla; **nu,** nuchal; **op,** opisthotic; **p1-2,** peripheral Plates 1-2; **pal,** palatine; **pt,** pterygoid; **q,** quadrate; **s,** scapula; **sq,** squamosal; **st,** stapes; **XII,** foramina of cranial nerve XII.

*Manchurochelys manchoukuoensis* Endo and Shikama [1] (Figures 2 and 3)

#### Referred specimen

PMOL-AR00180 (Figures 2 and 3), a partial articulated skeleton, including the skull, the first six cervical vertebrae, the anterior part of the carapace, two fragmentary scapulae, and a proximal end of the right humerus.

#### Locality and Horizons

The fossil is from a site near Qilinshan (Heishangou), Chifeng City, Inner Mongolia (E118°50′46.4″, N42°08′33.3″; Figure 1); the Early Cretaceous Jiufotang Formation [45]. Given the novelty of this site, detailed information is not yet available regarding its precise age or accompanying fauna.

#### Revised Diagnosis

*Manchurochelys manchoukuoensis* is diagnosed as a primitive pancryptodire by the presence of a low domed shell and a ligamentous connection between the plastron and carapace. It is distinguished from other basal pancryptodires by the following unique combination of characters: prefrontals contact one another along the midline, postorbital-squamosal contact absent, parietal and squamosal separated, crista supraoccipitalis relatively long, foramen palatinum posterius large, nuchal emargination shallow, cervical scale present, vertebral scales 2-4 longer than wide, first vertebral wider than nuchal, preneural absent, eight neurals present, peripheral 1 - costal contact present, costal 3 with parallel anterior and posterior sides, process or spine on peripheral 7 absent, two suprapygals present of which the posterior one is much larger than the anterior one, pygal present, central and posterior fontanelles absent, posterior lobe of plastron long and narrow.

### Description

#### Skull

The skull is exposed in dorsal and ventral views (Figures 2 and 3). The cranial elements can be readily distinguished from one another although some cracks are present due to diagenetic compression. The skull is slightly elongated and similar in its proportions to the skull of PMOL-AR00008. The skull roof is ornamented with a rugose surface and there are no apparent cranial scale sulci.

##### Dermal roofing elements

The nasals are not preserved, but were likely present by comparison to PMOL-AR00008. The dorsal plate of the right prefrontal is preserved, but its left counterpart is missing completely. The descending process cannot be observed on either side. Anteriorly, the right prefrontal is partially hidden by the right maxilla and the anterior contacts of the prefrontal with the adjacent elements are therefore uncertain. It seems that the prefrontals contact one another along the midline in PMOL-AR00180. In the description of PMOL-AR00008, the prefrontals were interpreted as being separated due to the anterior processes of the frontals [5]. However, a revision of this specimen reveals that the component of the frontal process that was actually exposed in the skull roof is short and did not separate the prefrontals completely.

Much of the frontal is well exposed except for the anterior process, which is only partially preserved on the left side. The anterior process is slender and a notch on its lateral side indicates an insertion for the prefrontal. Posterior to the notch, the frontal provides a small contribution to the dorsal rim of the orbit that is greater than that of PMOL-AR00008 [5]. More posteriorly, the frontal has a slightly curved suture that contacts the postorbital laterally. The frontal reaches its greatest width at the straight, posterior suture with the parietal.

The parietals are well exposed in dorsal view, forming an irregular pentagon in outline. They contact each other along their entire length, except for their distal ends, which are separated by the supraoccipital. On the skull surface the parietal contacts the frontal anteriorly, the postorbital laterally, and the supraoccipital posteriorly. Posterolaterally, the parietal contributes to the upper temporal emargination. As in *Sinemys* spp. the upper temporal emargination is well developed and the processus trochlearis oticum is therefore fully exposed in dorsal view. The deepest portion of the upper temporal emargination coincides with the parietal-postorbital suture. This condition is similar to that present in *Sinemys* spp. and *M. manchoukuoensis*, but contrast that present in *Ordosemys* spp., *Kirgizemys hoburensis*, and *Liaochelys jianchangensis*, where the parietal frames the deepest part of the upper temporal emargination by a distinct posterolateral process. The long and narrow processes of the parietals that surround the supraoccipital posteriorly are longer than those of PMOL-AR00008 ([5]: Figure 3ab), but this might be a preservational difference. The parietal has an additional lateral contact with the prootic within the upper temporal fossa.

The right jugal is preserved along the posteroventral corner of the fossa orbitalis. It has a long and slender anterior process that forms the ventral rim of the orbit together with the maxilla. Dorsally, the jugal has a curved sutural contact with the postorbital. Other, potential posterior contacts of the jugal with other elements are uncertain due to compression.

The presence of the quadratojugals is uncertain due to compression in the temporal area.

The squamosal is positioned at the posterolateral corner of the skull. A posteromedially directed low crest extends along the dorsal plate of the squamosal that frames the lateral aspects of the upper temporal emargination. The squamosal crest is short relative to the elongated crista supraoccipitalis and therefore similar to PMOL-AR00008. The posteriormost tip of the squamosal is pinched and directed posterolaterally, as in *K. hoburensis* and PMOL-AR00008. Medial to the crest, the squamosal contacts the quadrate anteriorly and the opisthotic posteromedially. The left squamosal is exposed in ventral view and reveals the anteromedial contacts with the quadrate and the opisthotic.

The right postorbital is preserved in articulation, whereas the left one is slightly offset from its original position. Anteriorly, the postorbital forms the posterior rim of the fossa orbitalis and contacts the frontal and parietal medially and the jugal laterally. The postorbital is the largest element in the temporal region and helps framing the deep upper temporal emargination together with the parietal.

##### Palatal elements

A slender and laminar fragment between the two maxillae is presumed to be the premaxilla.

The maxilla is the largest element of the snout. The vertical (prefrontal) process contacts the prefrontal dorsally and forms the lateral rim of the apertura narium externa and the anterior rim of the fossa orbitalis. The maxilla contacts the jugal posterodorsally. The horizontal (palatine) plate of the maxilla forms the triturating surface. The triturating surface consists of a longitudinal depression bordered by the labial ridge and a single lingual ridge. Both ridges are comparable in height along their anterior third, but the lingual ridge is distinctly higher than the labial one along its posterior third. The medial contact of the maxilla with the palatine is obscured in ventral view by the mandibles.

The vomer, an unpaired and elongate bone, is slightly displaced from its original position. Its contact with the adjacent elements is uncertain. The vomer is dumbbell-shaped with bilaterally expanded anterior and posterior ends and a keeled main body. The expanded ends are notched, the posterior notch being slightly less developed than the anterior one. These expansions coincide with Y-shaped divergences of the ventral, keel-like ridge of the main body. The posterior notch possibly received the anterior processes of the pterygoids.

The palatines are partially exposed in ventral view and slightly displaced from their original positions. The palatine is a flat plate that encloses the foramen palatinum posterius together with the maxilla and the pterygoid. The exact outline of the foramen palatinum posterius is unclear, but it was apparently large, as in *D. bicuspis* and *Sinemys* spp., which is different from the moderately sized condition seen in *Ordosemys* spp. and *Kirgizemys dmitrievi*. Posteromedially, the palatine has a broad and rounded edge to contact the vomer and the pterygoid.

##### Palatoquadrate elements

The quadrate is well exposed in dorsal and ventral view. It forms the wall of the cavum tympani. Within the temporal fossa, the quadrate has a broad sutural contact with the prootic medially and the squamosal posteriorly. Together with smaller contribution from the prootic, the quadrate forms a thickening at the anterior wall of the otic capsule, the processus trochlearis oticum. The trochlear process is poorly developed and therefore does not protrude significantly into the lower temporal fossa. The quadrate portion of the processus trochlearis oticum is sculptured along the prootic-quadrate suture by several small grooves and ridges, but it is unclear if these ridges had a particular function. In ventral view, the condylus mandibularis are well preserved on both sides of the skull except for a slight lateral twisting caused by compression. The articular surface is a concave facet. Posterior to the condylus mandibularis, a well-developed crest is apparent that runs parallel to the incisura columella auris. In many derived turtles, this crest contacts the posteroventral process of the quadrate to enclose the incisura columella auris. In PMOL-AR00180, however, such a contact is absent. However, this does not logically imply that the incisura was completely open posteriorly, since *Sinemys gamera* has a comparable morphology in ventral view but nevertheless exhibits a closed incisura in lateral view.

The pterygoid is a major element in ventral view. Anteriorly, the pterygoid has a short palatal process that is subtriangular and pointed rostrally. The pterygoids contact one another along their anterior thirds. More posteriorly, the pterygoids are separated from one another by the basisphenoid. Laterally, the pterygoid forms a horizontal plate with a concave posterior margin and a small, recurving processus pterygoideus externus. At the posterior margin of the skull, the pterygoid appears to have a contact with the basioccipital and exoccipital. The foramen posterius canalis carotici interni is not visible.

##### Braincase elements

The supraoccipital crest is notably elongated when compared to *Ordosemys liaoxiensis* or *Liaochelys jianchangensis* and reaches beyond the posterior tip of the squamosals, as in PMOL-AR00008. In lateral view, the crista supraoccipitalis has a slightly convex dorsal outline and a maximum height of approximately 3 mm. It contacts the parietals anterolaterally and the opisthotic and exoccipital laterally.

The exoccipitals are well exposed ventrally and are pierced by a pair of foramina nervi hypoglossi. More laterally, together with the opisthotic, the exoccipital encloses a large foramen, the foramen jugulare posterius, which is consistent with the condition seen in *Sinemys gamera.* The exoccipital has an anterior contact with the pterygoid. Medially, the exoccipital contacts the basioccipital and contributes to the condylus occipitalis.

The basioccipital forms the floor of the braincase together with the basisphenoid anteriorly and the exoccipitals posterolaterally. Anterolaterally, the basioccipital has a contact with the pterygoid. More posteriorly, the paired, horizontally oriented tubera basioccipitale are well developed and separated from one another by a deep midline depression. The distal portion of the basioccipital forms the ventral portion of the condylus occipitalis.

The prootic forms the processus trochlearis oticum together with the quadrate. The foramen stapedio-temporale is primarily enclosed by the prootic, but there is also a small contribution from the quadrate.

The opisthotic contacts the prootic anteriorly, the quadrate and squamosal laterally, and the supraoccipital and exoccipital medially. Posteriorly it encloses the foramen jugulare posterius together with the exoccipital.

The basisphenoid is pointed anteriorly and broadened posteriorly and therefore has a triangular outline. Anteriorly, it is wedged between the pterygoids. In comparison to closely related taxa, the basisphenoid appears to be greatly elongated, but this may be a result of damage to the pterygoids. Posteriorly, the basisphenoid contacts the basioccipital along a straight transverse suture and with the latter forms a smooth and flat braincase floor. The basisphenoid is sculptured by a round median depression, which may be a taphonomic artifact. The fenestra caroticus (sensu [36]) opens along the pterygoid-basisphenoid suture, anteriorly to the basisphenoid pits. Anteriorly, the fenestra ends in the foramen posterius canalis carotici cerebralis. The basipterygoid process of the basisphenoid and the foramen posterius canalis carotici palatinum are not visible, probably due to the displacement of the pterygoids.

The left columella auris (stapes) is well preserved with the basis columellae (footplate) and columella. As in modern turtles, the columella auris has a well-expanded basis columellae and a rod-like delicate columella. Medially, the basis columellae remains in situ and fits well into the fenestra ovalis, but this is partially obscured from ventral view by the hyoid bone. Laterally, the columella is well exposed between the fenestra ovalis and the quadrate, with a length of approximately 5.5 mm. The columella is broken at its middle point, and the distal part is offset slightly. However, its terminal end still remains within the incisura columella auris. The terminal end of the columella is slightly expanded.

#### Mandible

The mandibles are exposed in ventral view and form a gentle V-shaped outline. They are occluding with the skull. The left mandible appears to be nearly straight, while the right one appears to be convex, but this is likely a taphonomic artifact. Medially, the rami meet at a short, fused symphysis.

The hyoids consist at least of a pair of cornu branchiale I, of which the left one is incomplete distally. The cornu branchiale I is slender, elongated, and curved with a slightly expanded proximal end. Its length is approximately 22 mm. We now interpret the hyoid element preserved in PMOL-AR00008 as the cornu branchiale II because of its greater thickness and its greatly expanded distal end. This indicates that *Manchurochelys manchoukuoensis* probably possessed two pairs of ossified cornu branchiale even though these two pairs are not preserved in the available specimens.

#### Shell

Only the anterior part of the carapace is preserved, including the nuchal plate, first costal plates, first peripherals, left second peripheral, and a fragment of the left third peripheral (Figure 2). The shell appears to be low and is slightly sculptured by numerous tiny pits and grooves.

There is a shallow nuchal emargination, as in PMOL-AR00008 and *Liaochelys jianchangensis* but quite different from *Sinemys lens* and *Dracochelys bicuspis* where a deeper emargination is present. The nuchal is a massive element with a trapezoidal shape that contacts the first peripherals laterally and the first costal plates posteriorly. It is distinctly anteroposteriorly longer than that of *Sinemys brevispinus* (the morphology is not entirely clear in *S. lens*). The first costal plate shows a broad contact with the subtriangular first peripheral, as in PMOL-AR00008 and *Sinemys* spp., but unlike *Dracochelys bicuspis* where the first peripheral is triangular and does not contact the costal (Figures 2 and 3).

The sulci of the scales are clearly impressed on the carapace, including the cervical scale, the first vertebral scale, the anterior three marginal scales, and the first pleural scale. The cervical scale is present, as in PMOL-AR00008, but absent in *Sinemys lens* and *Dracochelys bicuspis*. It is small and sub-trapezoidal, with a maximum width of 15 mm and a minimum length of 4 mm. The first vertebral scale is hexagonal and distinctly wider than long. It contacts the cervical anteriorly, the first two marginals anterolaterally, and the first pleural posterolaterally. The first pleural is partially preserved on the left side. The anterior two marginal scales are identified on the right side, while the anterior three scales are present on the left side. The first marginal is much smaller than the second one.

#### Vertebral column

The cervical series is well preserved in articulation between the skull and shell, but only the six anterior cervicals can be identified with confidence. The articulation prohibits observing the development and orientation of the articular surfaces of the centra, and it is not possible to recognize cervical ribs along the cervical series. However, the centra are formed and the presence of a biconvex centrum can be excluded.

The atlas is displaced from its original position. The atlas neural arch is positioned against the supraoccipital and the left exoccipital and is slightly hidden by the latter anteriorly. The arch is clearly shorter than the axis. As in most crown cryptodires, the neural arch is a flat lamina. It is expanded dorsally to bear a broad medial contact with its counterpart. The neural arch bifurcates posteriorly with a short lateral spine and a medial process. The lateral spine is positioned slightly beyond the medial process. Medially, the spine conjoins the process along a semicircular notch. The medial process is broad for articulating with the prezygapophysis of the axis.

The axis is well preserved below the crista supraoccipitalis in articulation with the succeeding cervicals. As in crown turtles, the prezygapophyses of the axis face dorsolaterally, whereas the prezygapophyses of the following cervicals face dorsomedially. The axis is a large element with a length of 10 mm and a width of 8 mm, comparable to the following cervicals. In dorsal view, the axis is dumbbell-shaped due to the lateral expansion of the prezygapophyses and postzygapophyses. The prezygapophysis extends more anteriorly than laterally, thereby forming a dorsolaterally facing articular surface. In contrast, the postzygapophyses are expanded more laterally than posteriorly, thereby forming the maximum width of the axis. Between the postzygapophyses, there is a posterior notch with a gentle curvature. The neural spine is developed with a height of 1 mm, beyond the posterior notch. In ventral view, a well-developed keel is present along the entire length of the centrum. The keel is reduced posteriorly and disappears at the posterior margin of the centrum. The transverse processes are well developed, forming a maximum width of 10 mm. As in crown cryptodires, the transverse process is positioned along the anterior half of the centrum. Posteriorly, the centrum is compressed bilaterally.

The remaining cervicals are similar to each other in morphology. The third and fourth cervicals are well exposed in dorsal view. They are similar to the axis in general morphology except for the dorsomedially-facing prezygapophyses. The prezygapophyses are divergent laterally and comparable to the postzygapophyses in extent. The right prezygapophysis of the fourth cervical is flat and faces dorsally and medially. The anterior notch between the prezygapophyses is comparable to the posterior one between the postzygapophyses. This condition is different from PMOL-AR00008, in which the anterior notch has an angle of 120 degrees, and the posterior notch is anterior to the middle point of the neural arch with an angle of 70 degrees [5]. The neural spine is present on the whole length of the neural arch, different from *M. manchoukuoensis*, in which the neural spine is limited to the anterior half of the neural arch [5].

In ventral view, the third and fourth cervicals are comparable in size to the axis, while the fifth and sixth cervicals appear to be prolonged. The transverse process is positioned at the anterior portion of the centrum. Along the cervical series, the transverse process increases posteriorly in size. The posterior end of the centrum broadens posteriorly along the cervical series. The ventral keel is well developed along the whole length of the centrum. Posteriorly, the keel increases in depth along the cervical series.

#### Pectoral girdle

The scapulae are partially preserved on both sides in ventral view. The scapula is triradiate with a dorsally directed scapular process, a ventrally directed acromial process, and a laterally directed glenoid process. The scapular process is long and slender, but its distal end is hidden by the cervical series on both sides. The scapular process gradually expands proximally and forms a gentle curve with the acromial process. The scapular process is set at an angle of 87 degree relative to the acromial process. Most of the acromial process is broken on both sides. Its remains are slightly longer than the glenoid process on the right side and confluent proximally to the glenoid process and scapular process. In contrast, the glenoid process is stout and bears a laterally facing glenoid fossa. On the right side, the glenoid fossa is occupied by the humeral head. The left glenoid fossa is exposed with a concave facet. However, the precise configuration of the glenoid fossa is uncertain because the coracoid portion is missing.

#### Humerus

The proximal head of the humerus is partially preserved on the right side in articulation with the glenoid fossa. The lateral process is identifiable as a ventrally directed crest. Medially, there is a distinct intertubercular fossa between the lateral and medial processes. The medial process is expanded posteriorly and is larger than the lateral process.

## Results

### Phylogenetic Analysis

None of the four phylogenetic analyses placed xinjiangchelyids, sinemydids, or other Cretaceous basal eucryptodires within Testudines, the crown-group of turtles. However, the relationships of Cretaceous forms vary among three of the analyses. All four analyses agree in that all Cretaceous taxa are consistently placed in a derived position relative to Xinjiangchelyidae.

**Figure 4:**
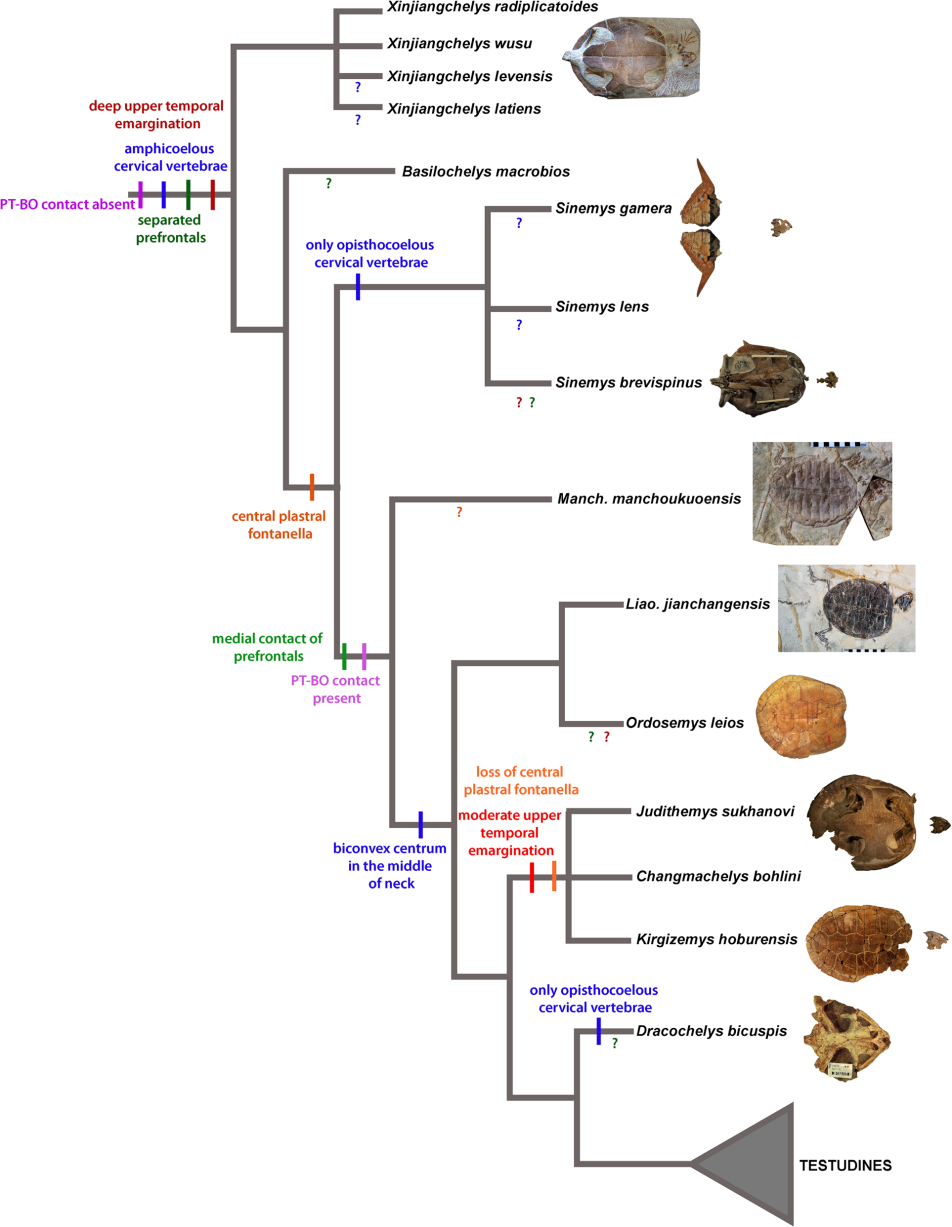
**Simplified strict consensus tree of Cretaceous basal eucryptodires retrieved from Analysis A (with no constraints and no new characters added) showing a paraphyletic arrangement for the taxa in question.** The inferred evolution of selected characters varying among these taxa is shown on the tree. Ambiguous character states for a given taxon are indicated with ‘?’ in a color that is corresponding to the color of that character. An alternative topology with *Changmachelys bohlini* not pruned from the strict consensus is shown in a box at the upper right corner.

*Analysis A* (220 equally parsimonious trees, tree length = 887): This analysis with no topological constraint and no new characters resulted in a largely paraphyletic arrangement of basal eucryptodires with a basally placed Sinemydidae (sensu [27]) that is only composed of *Sinemys* spp., an (*Ordosemys leios* + *Liaochelys jianchangensis*) clade and a successively more derived clade including *Judithemys sukhanovi*, *Kirgizemys hoburensis* and *Changmachelys bohlini* (the latter group roughly corresponds to the traditional circumscription of Macrobaenidae). *Manchurochelys manchoukuoensis* is found in the next less inclusive node to Sinemydidae. *Dracochelys bicuspis* occupies the most derived position among these taxa. The skull of *Ordosemys* sp. proved to be a wildcard taxon (Figure 4; Additional file 3).

*Analysis B* (136 equally parsimonious trees, tree length = 909): The constrained analysis obtained poor resolution for Cretaceous basal eucryptodires, similarly to the recent results of Rabi et al. [36]. However, a monophyletic Sinemydidae composed only of *Sinemys* spp. as well as a *Judithemys sukhanovi* – *Kirgizemys hoburensis* - *Changmachelys bohlini* clade was again recovered. Removal of wildcard taxa, including *Ordosemys* sp. and *Basilochelys macrobios* does not improve resolution (Additional file 3).

*Analysis C* (151 equally parsimonious trees, tree length = 925): Inclusion of new characters into the constrained analysis further decreases resolution. However, after pruning several Cretaceous taxa, a monophyletic Sinemydidae was obtained including *Sinemys* spp., *Manchurochelys manchoukuoensis*, and *Dracochelys bicuspis*. Removal of the constraint results in the basal placement of *M. manchoukuoensis* within Sinemydidae and in a (*Liaochelys jianchangensis* + *Ordosemys leios*) clade that in turn forms a polytomy with other Cretaceous basal eucryptodires (Additional file 3).

**Figure 5:**
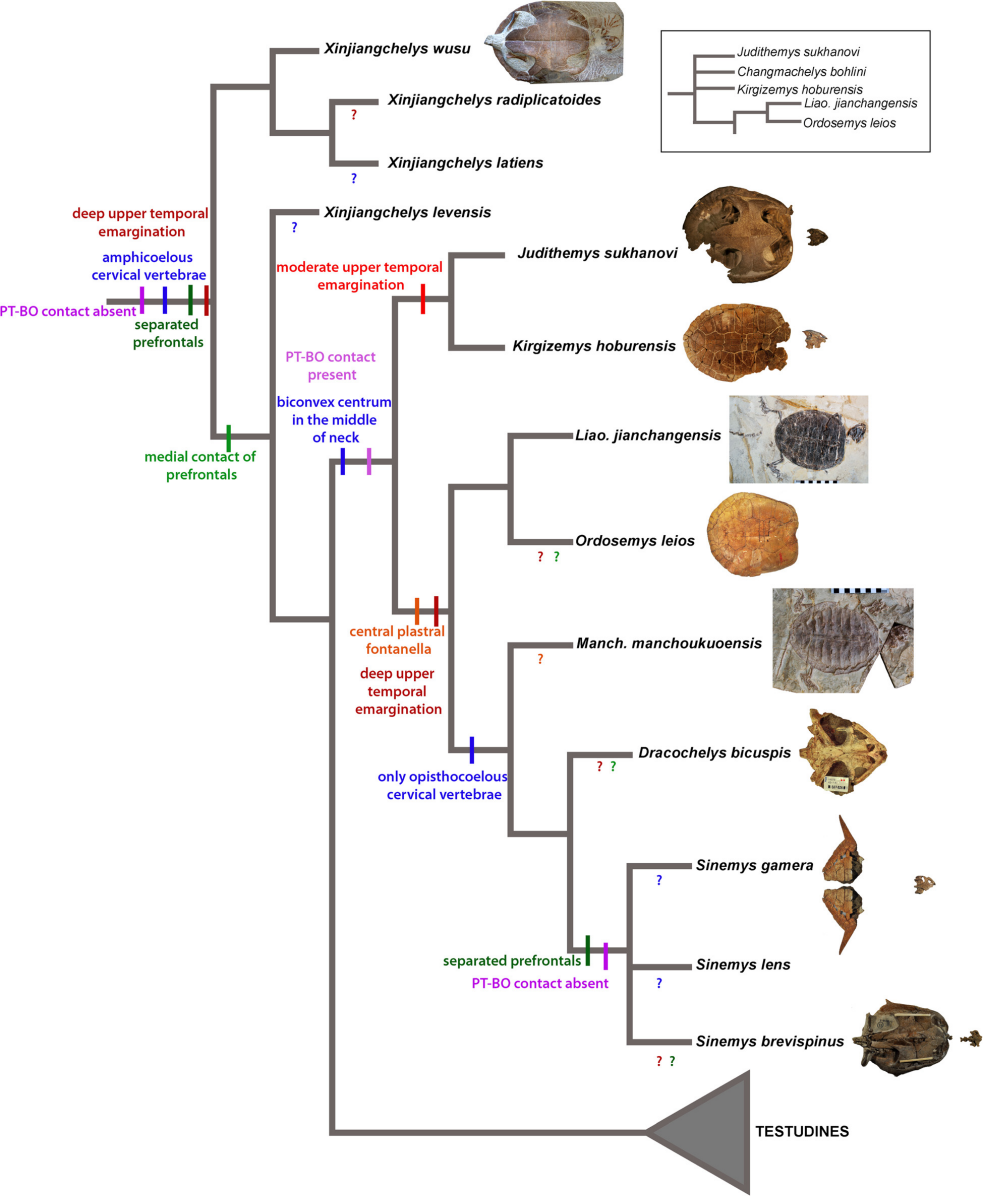
**Simplified strict consensus tree of Cretaceous basal eucryptodires retrieved from Analysis D.** In this analysis most pan-pleurodires and *Basilochelys macrobios* was a priori removed, nine new characters were added and the relationships of cryptodires were constrained according to molecular phylogenetic results. The inferred evolution of selected characters varying among these taxa is shown on the tree. Ambiguous character states for a given taxon are indicated with ‘?’ in a color that is corresponding to the color of that character.

*Analysis D* (143 equally parsimonious trees, tree length = 819): When most pleurodires and *Basilochelys macrobios* are *a priori* excluded from the analysis a monophyletic Sinemydidae (sensu [27]) is recovered containing all Cretaceous forms. *Manchurochelys manchoukuoensis* is sister to a (*Dracochelys bicuspis* + *Sinemys* spp.) clade and combined they are sister to an (*Ordosemys leios* + *Liaochelys jianchangensis*) clade. *Judithemys sukhanovi* + *Kirgizemys hoburensis* are the most basal sinemydids in the context of this analysis. *Ordosemys* sp. and *Changmachelys bohlini* turned to be acting as wildcards and the inclusion of any of them sinks the *J. sukhanovi* + *K. hoburensis* clade into a polytomy. Another notable feature of these results that *Xinjiangchelys* (= *Annemys*) *levensis* is no longer recovered as a xinjiangchelyid but in the next less inclusive node to them (Figure 5). Exclusion of new characters does not influence tree topology but decrease bootstrap support for the *Sinemys* spp. clade by 47%. Other changes in support are insignificant. A search without constraints results in a basal polytomy of Cretaceous basal eucryptodires with *Manchurochelys manchoukuoensis* placed as sister to *Sinemys* spp. when most other Cretaceous target taxa are pruned (Additional file 3).

## Discussion

### Taxonomic comments

PMOL-AR00180 is assigned to *Manchurochelys manchoukuoensis* because no major differences are apparent in the proportions and contacts in the skull and the shell with those of the holotype [1] or the referred specimen PMOL-AR00008 [5]. A striking similarity of PMOL-AR00180 with PMOL-AR00008 is the presence of a long supraoccipital crest that extends markedly more posteriorly than in *Liaochelys jianchangensis*, *Ordosemys liaoxiensis*, *Kirgizemys hoburensis*, and *Sinemys lens* (unknown for other species of *Sinemys*). Although differences appear to be present, at first sight, between the neck of PMOL-AR00008 and PMOL-AR00180, particularly in the degree of separation of the postzygapophyses, these are only because different sections of the neck are exposed in the two specimens. As in modern cryptodires, the postzygapophyses of the anterior cervicals in *M. manchoukuoensis* (as well as in *Sinemys brevispinus*, *Kirgizemys hoburensis*) are more fused than the posterior ones. In addition, the foramen posterius canalis carotici cerebralis was illustrated in the pterygoid in PMOL-AR00008 (labeled as foramen basisphenoidale [5]) but a revision of the specimen reveals that the position of this foramen is unclear and preservation makes comparison difficult with PMOL-AR00180 where it is on the pterygoid-basisphenoid suture.

With the discovery of the new specimen, the number of described *M. manchoukuoensis* fossils has increased to three. Two of these originate from the Yixian Formation of Liaoning Province (including the lost holotype [1,5]) whereas PMOL-AR00180 was recovered from the Jiufotang Formation of Inner Mongolia. Both formations are considered to be Lower Cretaceous, with the Yixian being Barremian to Upper Aptian (129-122 Ma) and the younger Jiufotang Formation being Aptian to Upper Albian (122-110 Ma) in age. Thus, the range of *M. manchoukuoensis* is extended geographically and, less unequivocally, temporally by the new fossil from Chifeng. However, given this difference in geography and perhaps in age it is not excluded that more complete findings may reveal distinct morphological features not preserved in the specimen from Chifeng and arguing for a separate species of *Manchurochelys*.

### Relationships of Cretaceous basal eucryptodire turtles

A broad range of phylogenetic hypotheses of Cretaceous basal eucryptodires has been proposed over the course of the last decade. Some studies consider these turtles to be a predominantly monophyletic clade [18,30,31,33] whereas others interpret them as being a predominantly paraphyletic assemblage [4,5,12,17,28,29,32,36]. Two analyses [20,34] obtained a monophyletic Sinemydidae to the exclusion of *Judithemys sukhanovi* and *Kirgizemys hoburensis*. A more crown-ward position for *J. sukhanovi* and *K. hoburensis* has been suggested in other studies as well [4,5,14,17,29,32,35]. All of these analyses build either on Gaffney [11] or Joyce [18]. The studies expanding the matrix of Gaffney [11] are problematic in assuming monophyly for many higher groups of turtles and for using a small number of characters only (max. 45), but they have the advantage of including many Cretaceous basal eucryptodiran taxa. The matrices expanding the work of Joyce [18] are improved in using single species as terminals and a large number of characters, but they are limited in taxon sampling, at least for the group in question. Other downsides of all of these analyses are the dominantly literature-based character scorings and the lack of specific, phylogenetically relevant characters for sinemydids, macrobaenids, and closely allied taxa.

We sought progress relative to previous analysis by significantly expanding the sample of Cretaceous basal eucryptodires, by utilizing a large, global matrix, by correcting several errors that are apparent in the scorings of previous matrices, through the addition of new characters relevant to this group, and by directly studying all relevant specimens. Therefore, our new analysis is the most comprehensive and the most exhaustive attempt to resolve the phylogeny of Cretaceous basal eucryptodires to date.

The results of our unconstrained phylogenetic analysis (Analysis A, Figure 4) agree in its primary aspects with the “paraphyletic hypothesis” of earlier global studies. However, the molecular backbone constraint of crown-cryptodire clades (Analysis B) collapses most of the nodes containing Cretaceous basal eucryptodires. Addition of new morphological characters places *Manchurochelys manchoukuoensis* and *Dracochelys bicuspis* within Sinemydidae together with *Sinemys* spp. while leaving other taxa largely unresolved. Interestingly, *a priori* exclusion of most panpleurodires and *Basilochelys macrobios* (Analysis D, Figure 5), results in the monophyly of all Cretaceous basal eucryptodires.

## Conclusions

The question remains unanswered whether the monophyletic or the paraphyletic hypothesis of basal eucryptodires is a better estimate of the phylogeny of sinemydid and macrobaenid turtles. From a parsimony point of view, the monophyletic arrangement is better supported since it requires five (or at least four, as two characters seem to be correlated) steps less than the paraphyletic topology as optimized within this part of the consensus trees of analysis A and D. However, most of these differences in the number of steps correspond either to the loss of traits, retention of juvenile characters (i.e. plastral fontanelles), or the acquisitions of highly variable and homoplastic characters (i.e. number of neurals). When only the acquisitions of more complex characters are taken into account, the differences are far less obvious and the monophyletic hypothesis appears to have less support. In this case, the monophyletic hypothesis requires a reversal to separated prefrontals (from medially contacting prefrontals) and the absence of pterygoid-basioccipital (and exoccipital) contact. On the other hand, the paraphyletic hypothesis requires that opisthocoely in the neck evolved twice within this group (Figures 4 and 5). In summary, until the position of panpleurodires relative to basal eucryptodires is instable, the phylogeny of sinemydids and macrobaenids remains ambiguous as well.

There are some other noteworthy results of the present contribution. The Mongolian *Kirgizemys hoburensis* and the North American *Judithemys sukhanovi* form a clade in all of our analyses (in agreement with some previous works; [17,29,34]) and the Chinese *Changmachelys bohlini* is part of the same clade in three of the analyses, though their exact relationships are unresolved. In addition, *Liaochelys jianchangensis* is found as the sister taxon of *Ordosemys leios* in three of the analyses. As for the target taxon of this work, *Manchurochelys manchoukuoensis* is placed within Sinemydidae (sensu [27]) together with *Sinemys* spp. and *Dracochelys bicuspis* in the majority of the analyses or alternatively, it is more derived than Sinemydidae and retained some typical characters of this group.

## Appendix 1

Changes to the taxon-character matrix of Rabi et al. [36]

Taxa added:

*Changmachelys bohlini*, *Manchurochelys manchoukuoensis*, *Liaochelys jianchangensis*, *Sinemys brevispinus*, *Sinemys gamera*, *Ordosemys* sp. skull [12]. Unlike in all previous analyses, *Ordosemys leios* is here treated separately from the *Ordosemys* sp. skull because they likely represent different species [12]. Most skull character scorings were therefore removed from *Ordosemys leios*, except for a few that could be deduced with the help of the skull fragments associated with the holotype.

Characters omitted from this study:

Maxilla B, Pterygoid M, Basioccipital B, Cervical Vertebra D, Cervical vertebra K (see explanation in ‘Methods’).

Characters modified in this study:

**Pterygoid I.** Vertical flange on processus pterygoideus externus: (0) absent; (1) present all along the process; (2) reduced.

*New definition:* Vertical flange of processus pterygoideus externus: (0) absent, (1) present.

The character of Sterli and de la Fuente [20] pertains to the vertical flange on the processus pterygoideus externus. This character is modified and completely rescored here because the definition of state 2 (reduced) is not clear and the original scorings of [20] do not help us to understand it either. For instance, *Emys orbicularis* and trionychids are scored as reduced in [20], whereas *Carettochelys insculpta* is not. In our opinion *Emys orbicularis* has a fully developed vertical flange, whereas *C. insculpta* and trionychids all have a reduced, barely thickened vertical component. Moreover, *Proganochelys quenstedti* and *Palaeochersis talampayensis* are scored 1 (vertical flange present almost all along the lateral process) but cheloniid sea turtle were scored as not having a vertical flange despite the clear presence of such a flange (which is more developed than in *P. quenstedti* and *Pal. talampayensis*). To assure better reproducibility we modify this character to contain only two states: vertical flange of processus pterygoideus externus: (0) absent or (1) present. Under the new definition *P. quenstedti*, *Kayentachelys aprix* and other basal turtles, including Meiolaniiformes, are scored as absent (0). And contrary to the previous scoring, we code *Carettochelys insculpta* and *Anosteira ornata* as present (1) because we see no difference from the trionychid condition.

**Cervical Vertebra I.** Sterli and de la Fuente [20] accidentally used “posteroventrally” not “anteroventrally” for the direction of the postzygapophyses of the 8^th^ cervical. *Caretta caretta* and *Chelonia mydas* is changed from 0 to 1 because they clearly show posteroventrally directing postzygapophyses.

Character rescored in this study:

**Pterygoid L.** Processus pterygoideus externus: (0) like in *Proganochelys quenstedti*; (1) like in testudinoids; (2) like in *Kayentachelys aprix*. This character pertains to the outline of the processus pterygoideus externus. In state 0 the processus is reduced, in state 1 it is better developed whereas in state 2 it is posteriorly recurved. The character is completely rescored because Sterli and de la Fuente [20] accidentally scored testudinoids with state 2.

*Changes to the character scorings* of Rabi et al. [36] (for justification see the corresponding character in the taxon-character matrix (Additional file 1).

Prefrontal A: *Dracochelys bicuspis*: 1—›?

Prefrontal D: *Dracochelys bicuspis*: 1—›?

Prefrontal E: *Dracochelys bicuspis*: 1—›?

Parietal A: *Dracochelys bicuspis*: 1—›?

Parietal G: *Dracochelys bicuspis:* ?—›1 ; *Kirgizemys hoburensis:* ?—›1

Parietal H: *Dracochelys bicuspis:* 2—›?; *Ordosemys* sp. skull: 2—›1

Jugal A: *Dracochelys bicuspis:* 1—›?; *Ordosemys* sp. skull: ?—›1

Quadratojugal A: *Sinemys lens*: 0—›?

Premaxilla A: *Xinjiangchelys wusu*, *Xinjiangchelys* (= *Annemys*) *levensis*, *Xinjiangchelys radiplicatoides*: 0—›1

Premaxilla E: *Ordosemys* sp. skull: ?—›0

Maxilla A: *Ordosemys* sp. skull: ?—›0

Maxilla B: *Kirgizemys hoburensis*: 1—›0

Maxilla C: *Ordosemys* sp. skull: ?—› –

Maxilla D: *Ordosemys* sp. skull: ?—›0; *Kirgizemys hoburensis*: ?—›0

Quadrate D: *Sinemys lens*: 0—›?

Quadrate F: *Sinemys lens*: 2—›?

Quadrate H: *Sinemys lens*: 1—›0

Epipterygoid A: *Kirgizemys hoburensis*: ?—›1

Pterygoid D: *Kirgizemys hoburensis*: 1—›0/1

Pterygoid G: *Kirgizemys hoburensis*: 1—›0

Pterygoid J: *Judithemys sukhanovi*: ?—›1

Supraoccipital A: *Dracochelys bicuspis*: 1—›?

Basioccipital A: *Kirgizemys hoburensis*: 1—›0

Basisphenoid A: *Kirgizemys hoburensis*: ?—›0

Basisphenoid B: *Dracochelys bicuspis*: ?—›0

Basisphenoid E: *Kirgizemys hoburensis*: ?—›0

Hyomandibular Nerve A: *Xinjiangchelys radiplicatoides*: 1—›0

Foramen Jugulare Posterius A: *Judithemys sukhanovi*: ?—›1; *Kirgizemys hoburensis*: ?—›0

Foramen Nervi Hypoglossi: *Xinjiangchelys wusu*: 2—›0; *Xinjiangchelys radiplicatoides*: 2—›0

Fenestra Perilymphatica A: *Sinemys lens*: 0—›?

Cranial Scutes A: *Judithemys sukhanovi*: 1—›?

Dentary A: *Kirgizemys hoburensis*: ?—›0

Carapace D: *Judithemys sukhanovi*: ?—›0

Nuchal C: *Kirgizemys hoburensis*: ?—›0

Neural B: *Kirgizemys hoburensis*: ?—›1

Cervical A: *Dracochelys bicuspis*: ?—›1

Marginal A: *Kirgizemys hoburensis*: ?—›0

Entoplastron D: *Dracochelys bicuspis*: ?—›0

Entoplastron F: *Kirgizemys hoburensis*: 0—›1

Epiplastron A: *Sinemys lens*: – —›0

Cervical Rib A: *Kirgizemys hoburensis*: ?—›0

Cervical Vertebra B: *Kirgizemys hoburensis*: 0—›1

Cervical Vertebra C: *Kirgizemys hoburensis*: ?—›1; *Judithemys sukhanovi*: ?—›1

Cervical Vertebra G: *Sinemys lens*: 0—›?

Cervical Vertebra H: *Kirgizemys hoburensis*: ?—›1

Cervical Vertebra I: *Kirgizemys hoburensis*: ?—›1; *Judithemys sukhanovi*: 0—›0&1

Caudal B: *Kirgizemys hoburensis*: ?—›1

Pectoral Girdle A: *Kirgizemys hoburensis*: ?—›1

Cleithrum A: *Kirgizemys hoburensis*: ?—›2; *Judithemys sukhanovi*: ?—›2

Scapula A: *Kirgizemys hoburensis*: ?—›2

Humerus C: *Kirgizemys hoburensis*: ?—›0

Humerus D: *Kirgizemys hoburensis*: ?—›0

Humerus E: *Kirgizemys hoburensis*: ?—›1

Illium A: *Kirgizemys hoburensis*: ?—›1

Pes A: *Dracochelys bicuspis*: – —›0

## Appendix 2

Definitions of new morphological characters:

**Posterior Plastral Fontanelle:** posterior plastral fontanelle between the xiphiplastra and/or the hypoplastra: (0) absent in adult stage; (1): retained in adult stage.

**Neural Number:** number of neurals (0) less than 9 elements; (1) nine elements.

**Plastron Lobe:** posterior lobe of plastron (0) relatively wide and short; (1) posterior lobe of plastron elongated and narrow coupled with widely spaced plastral buttresses.

*Comment:* This character is included to capture the characteristic proportions of the posterior lobe of *Manchurochelys manchoukuoensis*, *Sinemys lens* and *Sinemys brevispinus* (unknown for *S. gamera*). In these taxa the posterior lobe is not simply just long and narrow with subparallel lateral sides but the base of the hyo- and hypoplastral buttresses are also placed wide apart resulting in an extensive central part of the plastron. Both these criteria have to be fulfilled in the derived state.

**Shape of Costal 3:** costal 3 (0) tapering towards the lateral side of the shell or with parallel anterior and posterior borders; (1) costal 3 broadens towards the lateral side of the shell. *Comment:* this character is shared by *Dracochelys bicuspis* and *Liaochelys jianchangensis* (especially marked in the latter).

**Costal Rib:** (0) distal portion of costal ribs not visible within the costal; (1) distal portion of costal rib visible on the surface of the costal.

*Comment:* In *Liaochelys jianchangensis* and *Dracochelys bicuspis* the rib portion of the costal is visible distally within the costal element. This is not to be confused with the presence of free rib heads in taxa with peripheral fontanelles which is a much more widespread character. With this character the rib has to be visible on the surface of the costals.

**Carapacial Sutures:** (0) carapacial elements finely sutured or the contact is smooth; (1) carapacial sutures strongly serrated in adult stage.

*Comment:* This character is shared by *Liaochelys jianchangensis* and *Dracochelys bicuspis* whereas other Cretaceous pancryptodires have smooth contacts between the elements of the carapace.

**First Vertebral:** (0) vertebral 1 does not enter anterior margin of carapace; (1) enters anterior margin.

*Comment:* the derived state is present in *Dracochelys bicuspis* (based on [37]), *Sinemys* spp. (unknown in *Sinemys gamera*) and curiously also in the aberrant pleurodire, *Araripemys barretoi* Price [46].

**Peripheral Gutter:** (0) peripheral gutter absent or only anteriorly developed; (1) peripheral gutter extensively developed along anterior and bridge peripherals. *Comment:* extensive gutter along the anterior two-thirds of the peripheral ring is characteristic for a number of Mesozoic pancryptodires [47,48] but has never been used in a phylogenetic analysis.

**Costal Rib Distal End:** (0) distal end of dorsal rib not visible or only within costo-peripheral fontanelles on the dorsal face of the carapace; (1) distal end of posterior dorsal ribs visible and surrounded by the peripheral. Comment: In sinemydids and a number of other related taxa the distal end of the posterior dorsal ribs are exposed in dorsal view within the peripherals even though these forms lack costo-peripheral fontanelles.

## Abbreviations

IVPP: Institute of Vertebrate Paleontology and Paleoanthropology, Beijing, China; PIN: Paleontological Institute, Russian Academy of Sciences, Moscow, Russia; PMOL: Paleontological Museum of Liaoning, Shenyang Normal University, Shenyang, China; TMP: Royal Tyrrell Museum of Palaeontology, Drumheller, Canada.

## Competing interests

The authors declare no competing interests.

## Authors’ contributions

CFZ initiated study, described and illustrated specimens. MR and WGJ collected data and MR performed analyses. MR, CFZ and WGJ wrote the manuscript. All authors read and approved the final manuscript.

## Supplementary Material

Additional file 1 Taxon-character matrix in nexus format. Click here for file

Additional file 2 Taxon-character matrix in tnt format. Click here for file

Additional file 3 Strict consensus trees. Click here for file
