# Supplementary material for: A new specimen of Manchurochelys manchoukuoensis from the Early Cretaceous Jehol Biota of Chifeng, Inner Mongolia, China and the phylogeny of Cretaceous basal eucryptodiran turtles
Source: BMC Evol Biol. 2014 Apr 5;14:77. doi: 10.1186/1471-2148-14-77 (PMC4021230; doi:10.1186/1471-2148-14-77)

ADDITIONAL FILE 3

Consensus trees for the phylogenetic analyses of Zhou, Rabi and Joyce: “A new specimen of *Manchurochelys manchoukuoensis* from the Early Cretaceous Jehol Biota of Chifeng, Inner Mongolia, China and the phylogeny of Cretaceous basal eucryptodiran turtles”. *BMC Evolutionary Biology.*

Pruned strict consensus tree of Analysis A

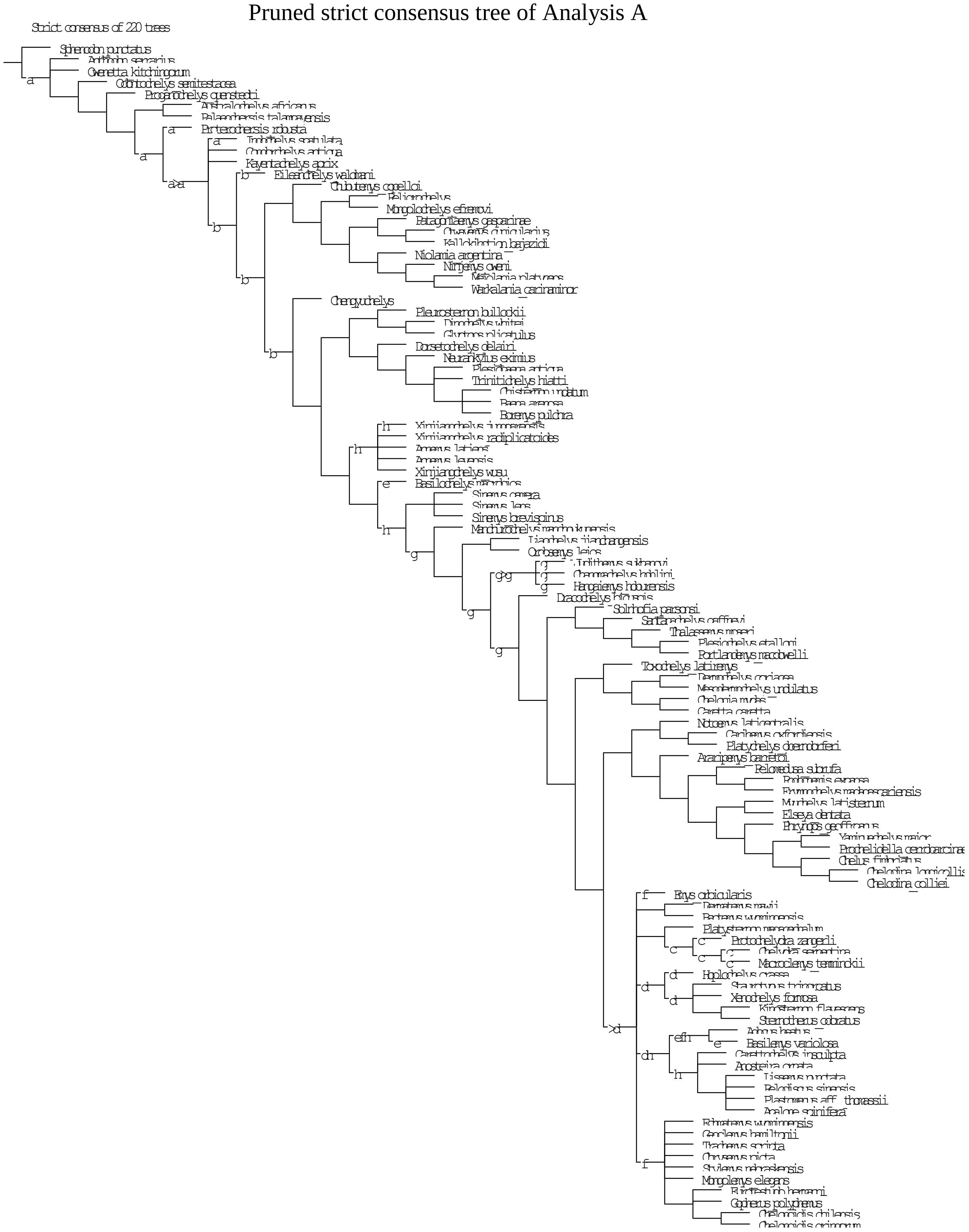

Strict consensus tree of Analysis B

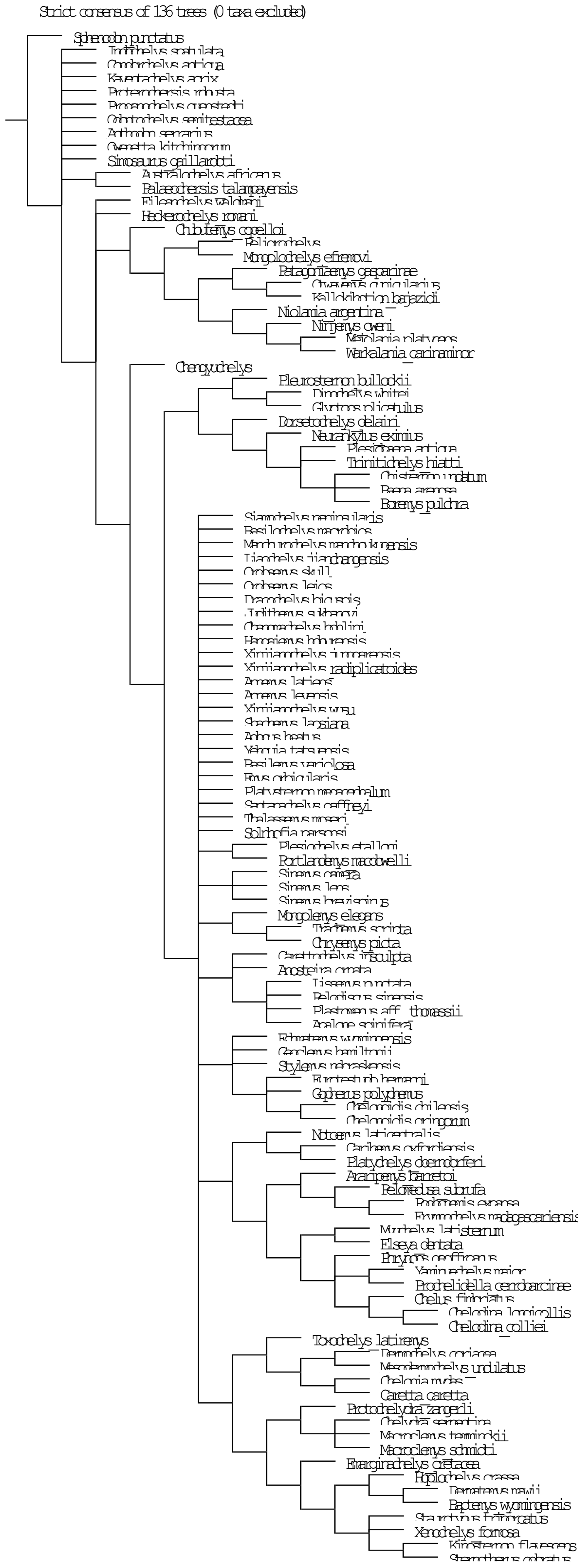

Pruned strict consensus tree of Analysis B

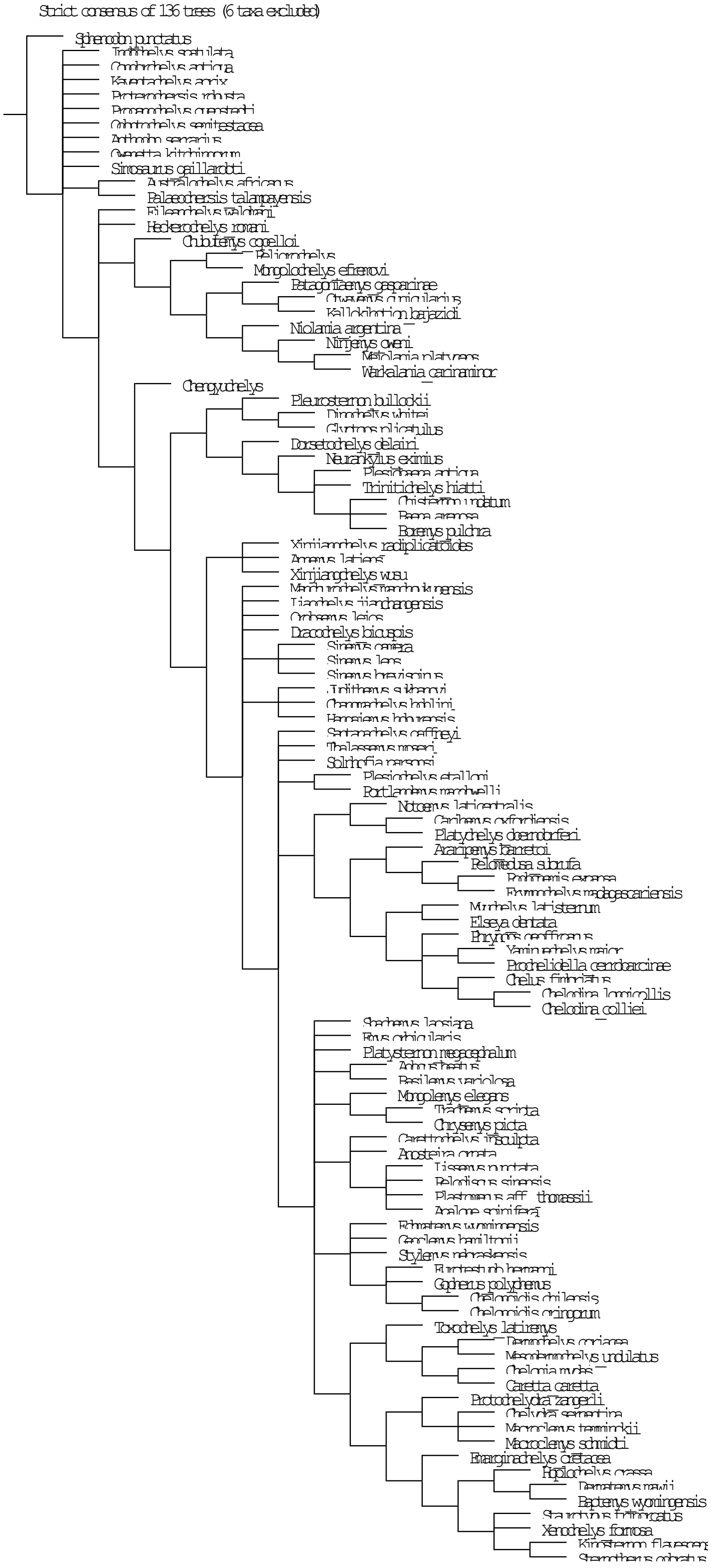

Strict consensus tree of Analysis C

Strict consensus of 151 trees (0 taxa excluded)

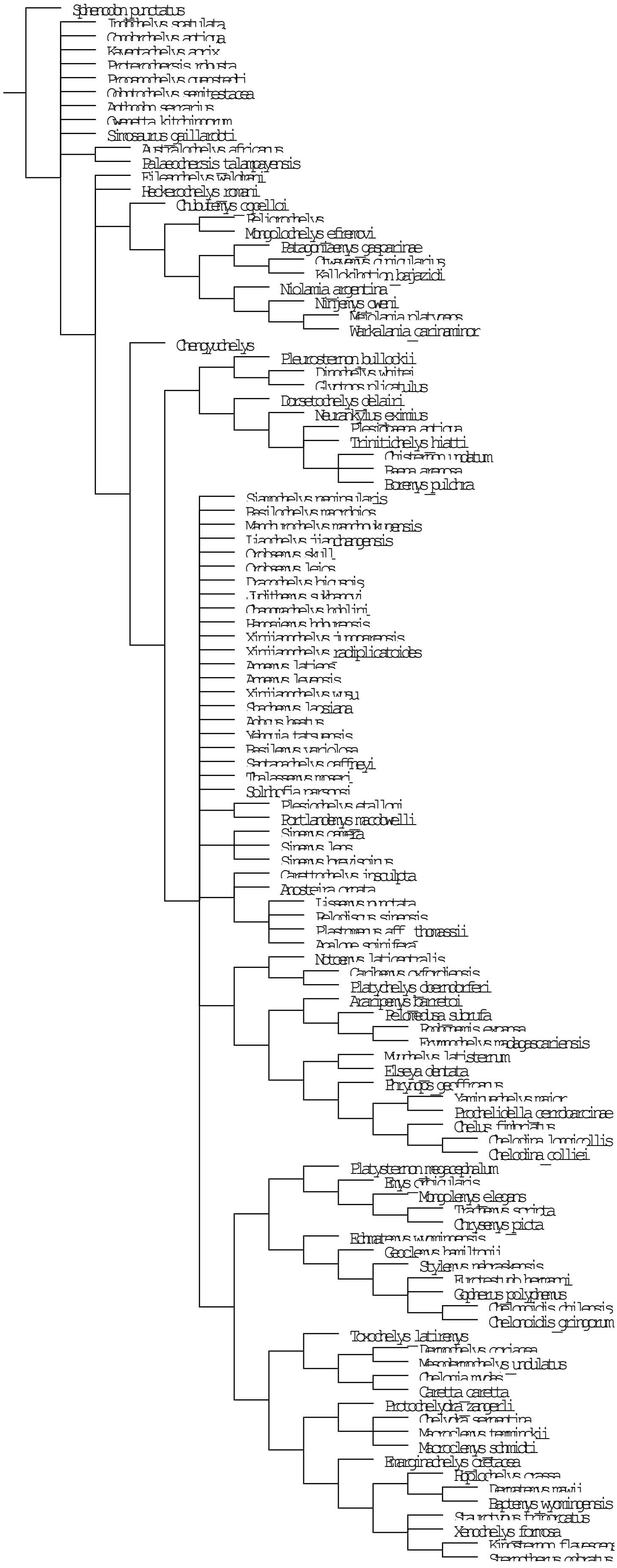

Pruned strict consensus tree of Analysis C

Strict consensus of 151 trees

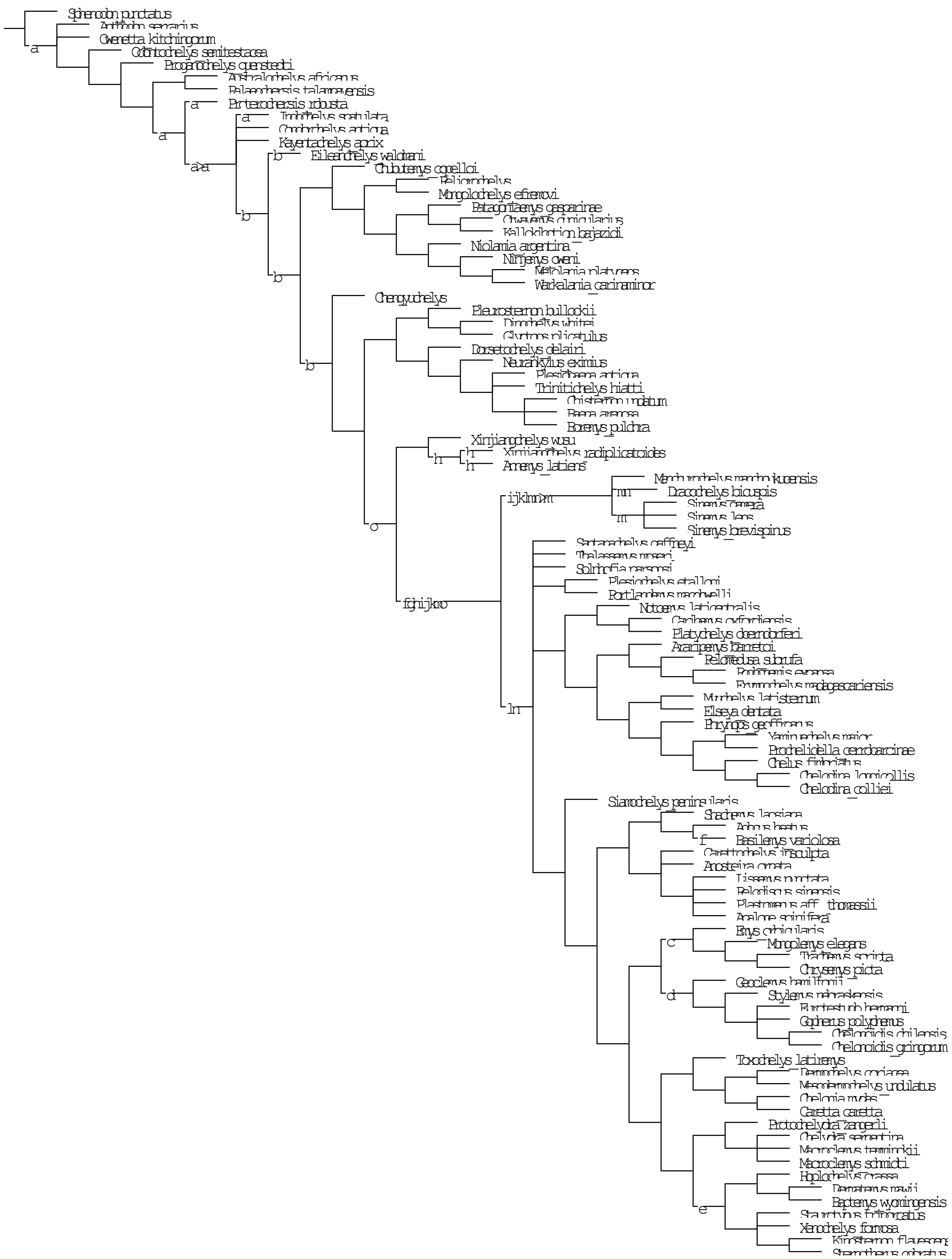

Strict consensus tree of Analysis D

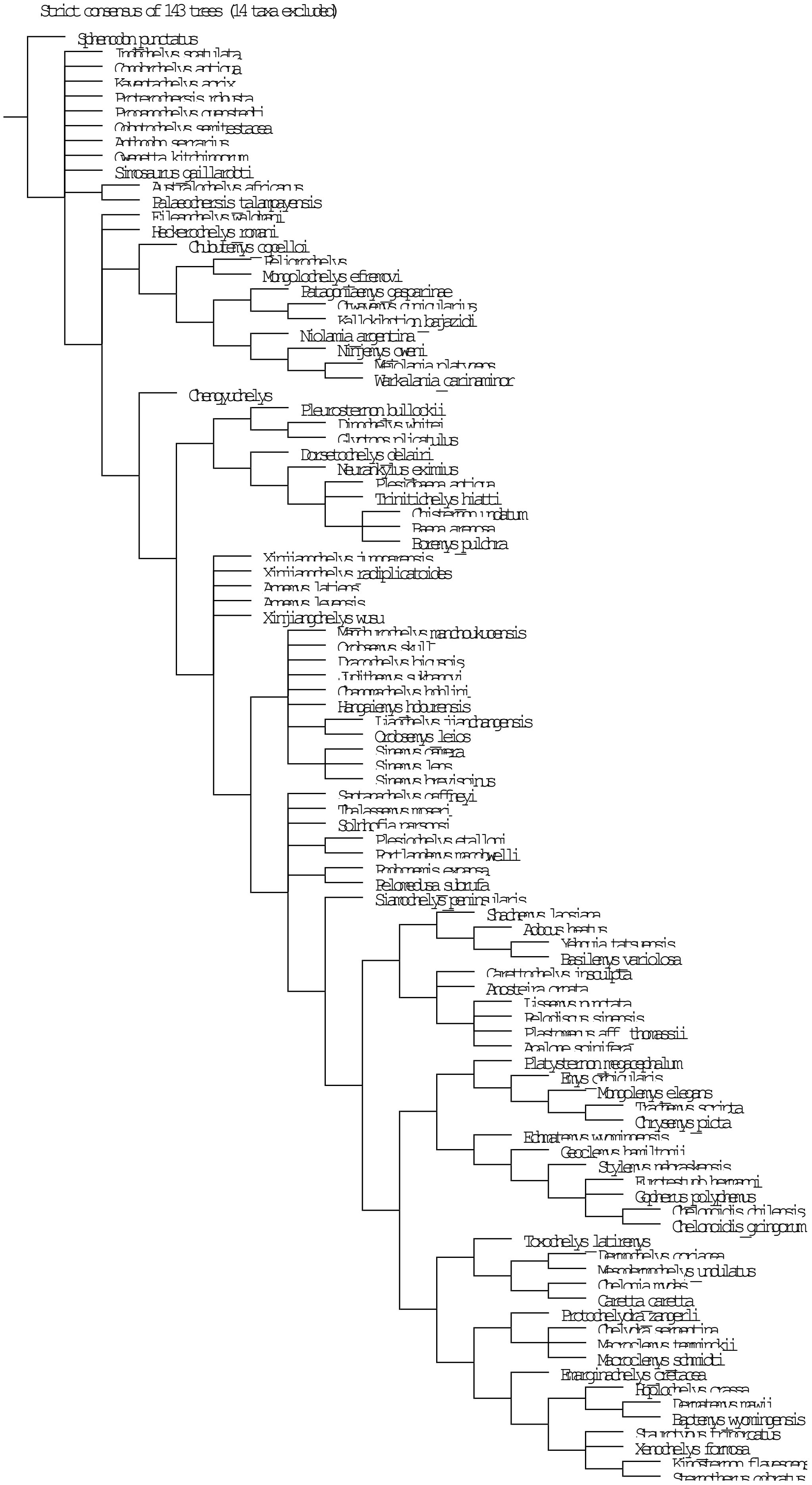

Pruned strict consensus tree of Analysis D

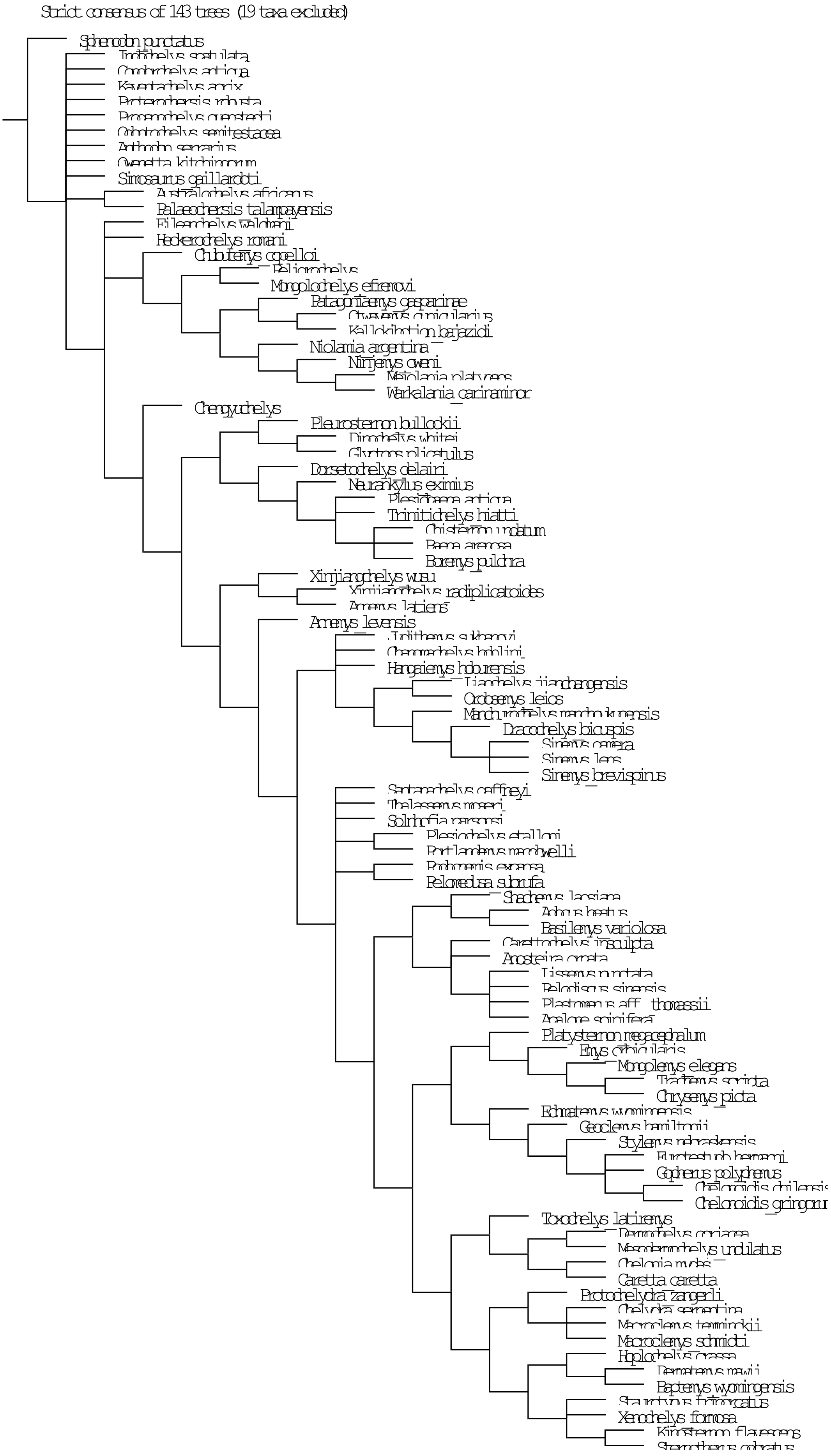

Bootstrap values for Analysis A

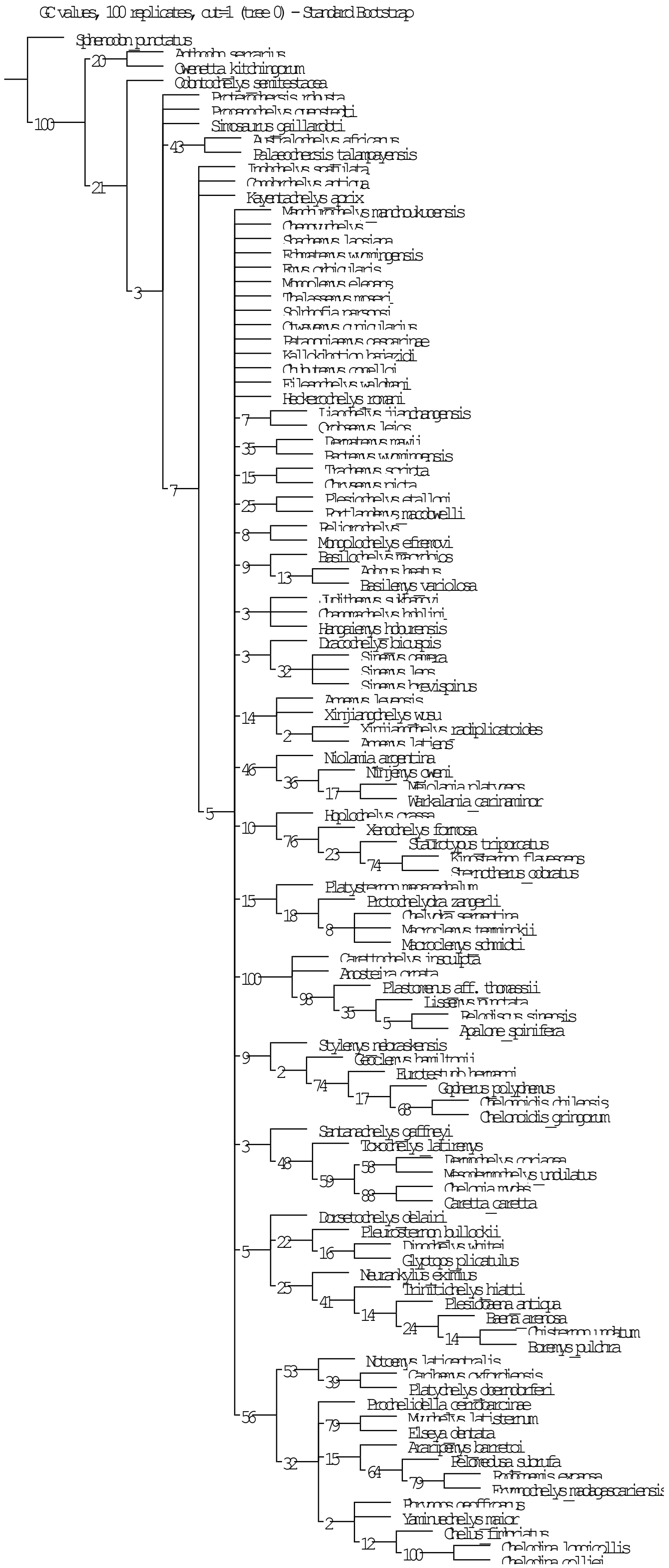

Bootstrap values for Analysis D

GC values, 100 replicates, cut=1 (tree 1) - Standard Bootstrap

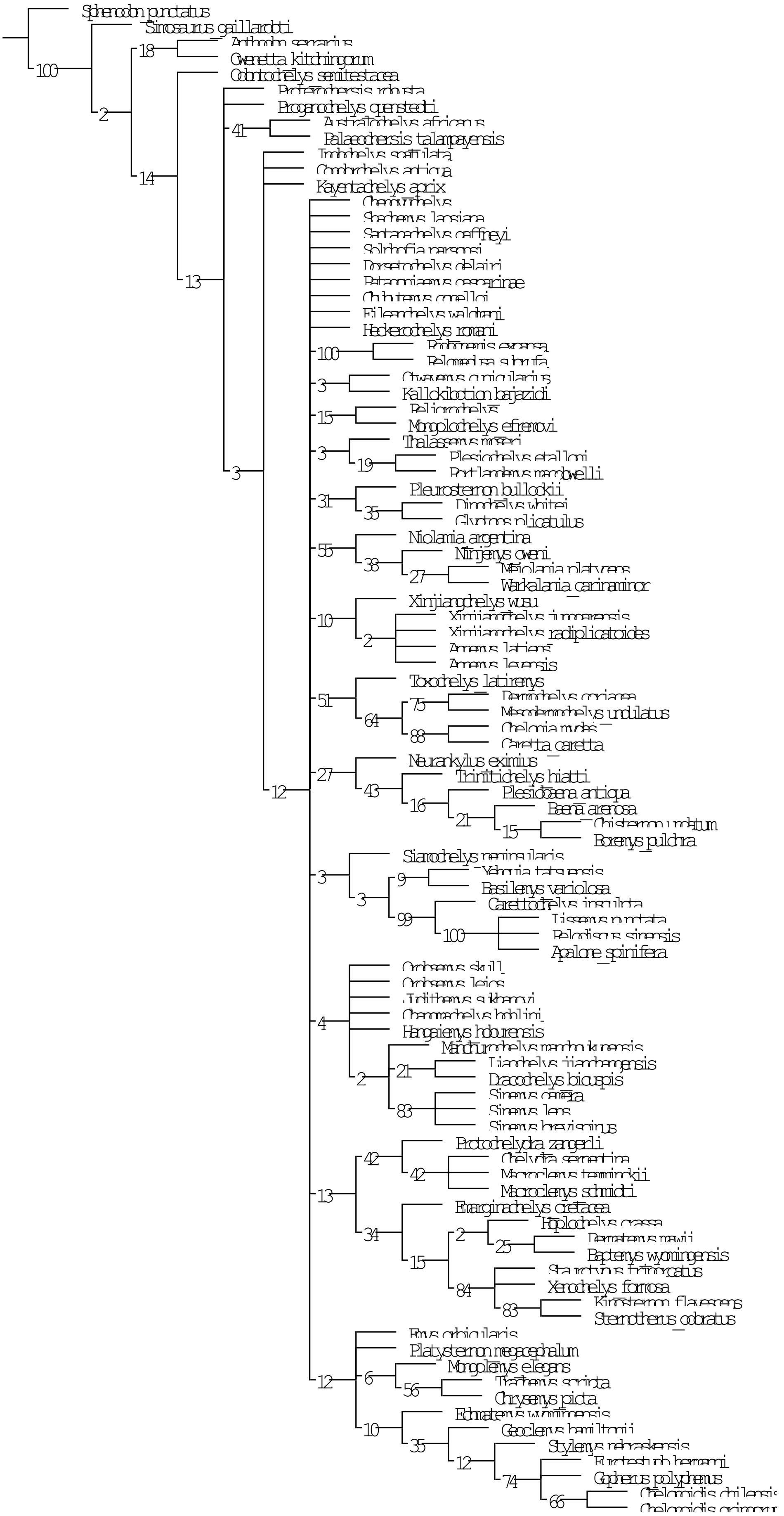

Bootstrap values for Analysis D with new characters excluded

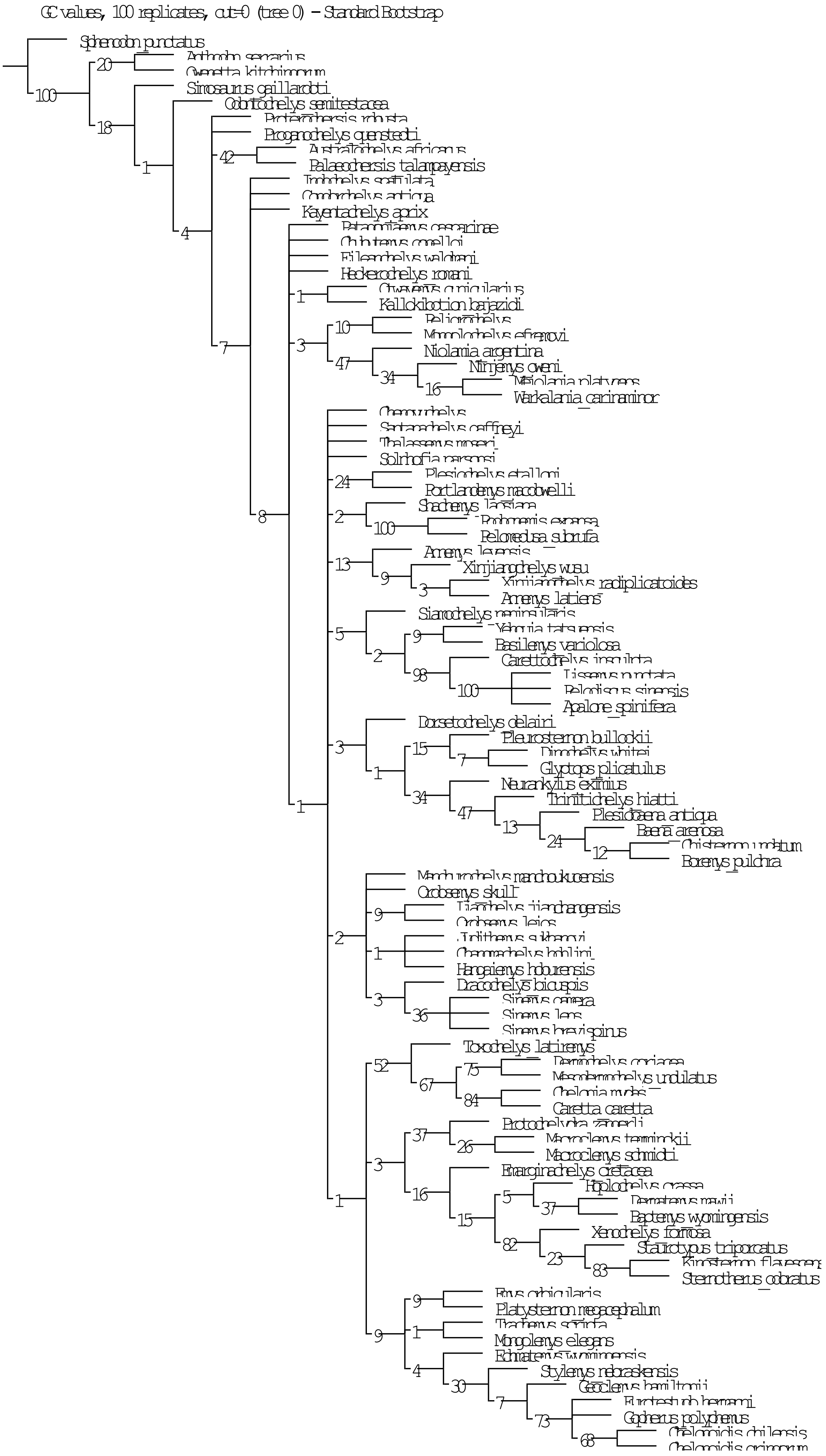

Supplement: Additional file 3 — Strict consensus trees. [file 1471-2148-14-77-S3.pdf]
